# Ethnobotanical investigation of medicinal plants in Buska Mountain range, Hamar district, Southwestern Ethiopia

**DOI:** 10.1186/s13002-022-00558-0

**Published:** 2022-09-19

**Authors:** Melese Bekele, Feleke Woldeyes, Ermias Lulekal, Tamrat Bekele, Sebsebe Demissew

**Affiliations:** 1 Ethiopian Biodiversity Institute, P.O. Box 30726, Addis Ababa, Ethiopia; 2 Department of Plant Biology and Biodiversity Management, Addis Ababa University, P.O. Box 3434, Addis Ababa, Ethiopia

**Keywords:** Buska Mountain range, Conservation, Ethnobotany, Indigenous knowledge, Local community, Medicinal plants

## Abstract

**Background:**

Despite the fact that ethnobotanical studies have been conducted in various parts of Ethiopia, compared with the existence of the multitude and diverse ethnic groups and their associated traditional knowledge, the studies are not comprehensive enough for all the localities and communities in the country. This is also true for the Hamar community of Southwestern Ethiopia, who are totally dependent on plants and plant products for their livelihood. Hence, this investigation was done to identify and record medicine plants and the native wisdom of the community in the area.

**Methods:**

Three hundred twenty six (326) informants were selected from the 12 lowest governmental units (Kebeles) applying Cochran’s formula through stratified random sampling technique. From the total informants, 74 (48 males and 26 females) were purposively selected for in-depth discussion. Semi-structured interviews, focus group discussions, guided field walks as well as market surveys were used for data collection. Standard ethnobotanical analytical tools comprising ranking and comparison were used for the analysis. Preference ranking, pair-wise comparison, informant consensus factor, direct matrix ranking, Cultural Significance Index (CSI) and Jaccard’s similarity coefficient (JCS) as well as Analysis of Variances (ANOVA), applying SPSS (version 20) were computed.

**Results:**

A total of 145 species practical to cure about 72 ailments of livestock and humans were recorded. Families Fabaceae (with 22% of species), Asteraceae (11%), and Lamiaceae (9%) were discovered as the most dominant families in the area. Shrubs contributed the most (40%) to the growth forms followed by herbs (26.5%). Fresh leaves of the plants were parts that are used most frequently in the area. The highest ICF value (0.94) was recorded for reproductive problem categories. There was a relatively very high dependence of the community on plants and plant products together with a hoarded indigenous knowledge in the area that positively correlated with age (*r* = 0.82).

**Conclusion:**

The study's findings revealed that Buska Mountain range is a home for highly diverse and most dependable plant species and associated indigenous knowledge. However, because of the realized environmental threats in the area, the conservation efforts of the community should be invigorated and supported in order to sustain the biodiversity in general and the medicinal plant species in particular.

**Supplementary Information:**

The online version contains supplementary material available at 10.1186/s13002-022-00558-0.

## Introduction

Over centuries, indigenous peoples from various parts of the world have established their own unique knowledge of plants which are relatively closer to their natural and agricultural environments [1, 2]. Uses of plants for food, household, utensils, medicine, building, clothing, magic, rituals, firewood, musical instruments, pesticides, shelter, cosmetics, divination, dyeing, textiles, tools, ornamentation, currency, and social life are all investigated in ethnobotany [3–5].

Ethnobotanical assessment of medicinal plants aims to explore and document a society's knowledge pertinent to traditional utilization of medicinal plants in their cultural settings [1, 6]. The world health organization [7] defined traditional medicine as health practices approaches, knowledge, and beliefs that include plant, animal, and mineral-based medicines, spiritual therapies, manual techniques, and exercises that are used singly or in combination to treat, diagnose, and prevent illnesses, as well as to maintain well-being. Indigenous peoples, particularly in developing countries, rely on plants for food, medicine, energy, and material culture [8]. Traditional medicine comprised of therapeutic practices that have been in existence for hundreds of years, before the development and delivery of modern medicine and are in use today [9]. However, current rises in population growth resulted in extensive forest clearing for expansion of agriculture in marginal areas, and hence lead to a subsequent loss of important plants in the wild and their associated knowledge [10]. The traditional medicine use customs of the communities may vary greatly based on the social and cultural heritages of the nations.

The Ethiopia’s geographical position, complex topography, environmental heterogeneity, ecological conditions, range of terrestrial and aquatic ecosystems offered suitable environment for a wide range of life-forms [11–13]. As a result, Ethiopia is home to a diverse range of languages, cultures, and beliefs, all of which have contributed to the diversity of traditional knowledge and practices of the people, as well as distinct ways of utilizing the country's flora [14, 15]. In the country, almost 80% of the human population and 90% of livestock rely on plant products for medicine, which are culturally rooted in all communities [16]. Because of the numerous circumstances including accessibility, economic availability, cultural acceptability and efficacy, medicinal plants are in use for millennia across various communities. Several tribal groups in Ethiopia have fascinating traditions that are based in large part and rich in plant knowledge. Communities of pastoralists and agro-pastoralists on dry land area, in particular, have the indigenous knowledge needed to use plant products for medicine, economic, cultural, and environmental purposes [17].

It is particularly relevant to the people in the South Omo Zone, who are fully reliant on plants and plant products for human and livestock. Specifically, the ethnobotanical practice of Hamar community in the Buska Mountain range has remained the least explored for scientific study due to the remoteness of the area and arid climatic conditions. The community is exclusively dependent on plants and plant products, though the ethnobotanical documentation is yet scant. As a result, this study was conducted with the following specific objectives: (1) to investigate the distribution, abundance and taxonomic diversity of medicinal plants (2) to document the traditional medicinal plants lore of the Hamar community and (3) to determine the threating factors to medicinal plants in the area.

## Methods and materials

### Study area

**Fig. 1:**
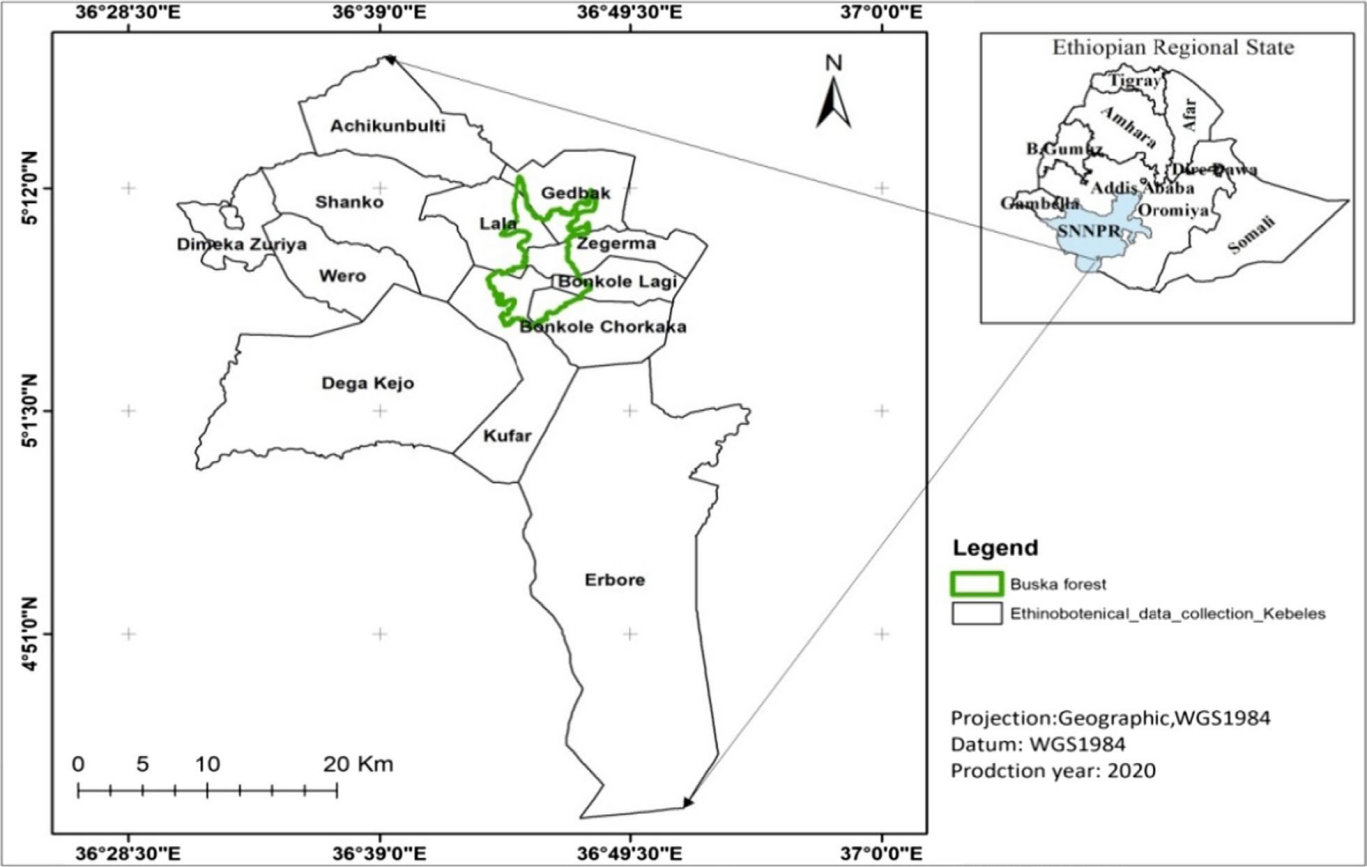
Map of Ethiopia showing the vegetation of Buska Mountain range and the study sites

The Hamar district is one of the eight districts in South Omo Zone, with coordinates ranging from 5° 12′ 40ʺ N latitudes and 36° 20′ 10ʺ E longitude. It borders to north by the Bena-Tsemay district, to the west by the Nyangatom district, to south by the Dasenech district, and to the east by the Borena rangeland of the Oromia region. The research location is at about 880 km south of Addis Ababa (Capital City of Ethiopia). The altitudinal range of the districts varies from 380 m.a.s.l. in the Erbore lowland to 2100 m.a.s.l. at the Peak of Buska Mountain (Fig. 1).

**Fig. 2:**
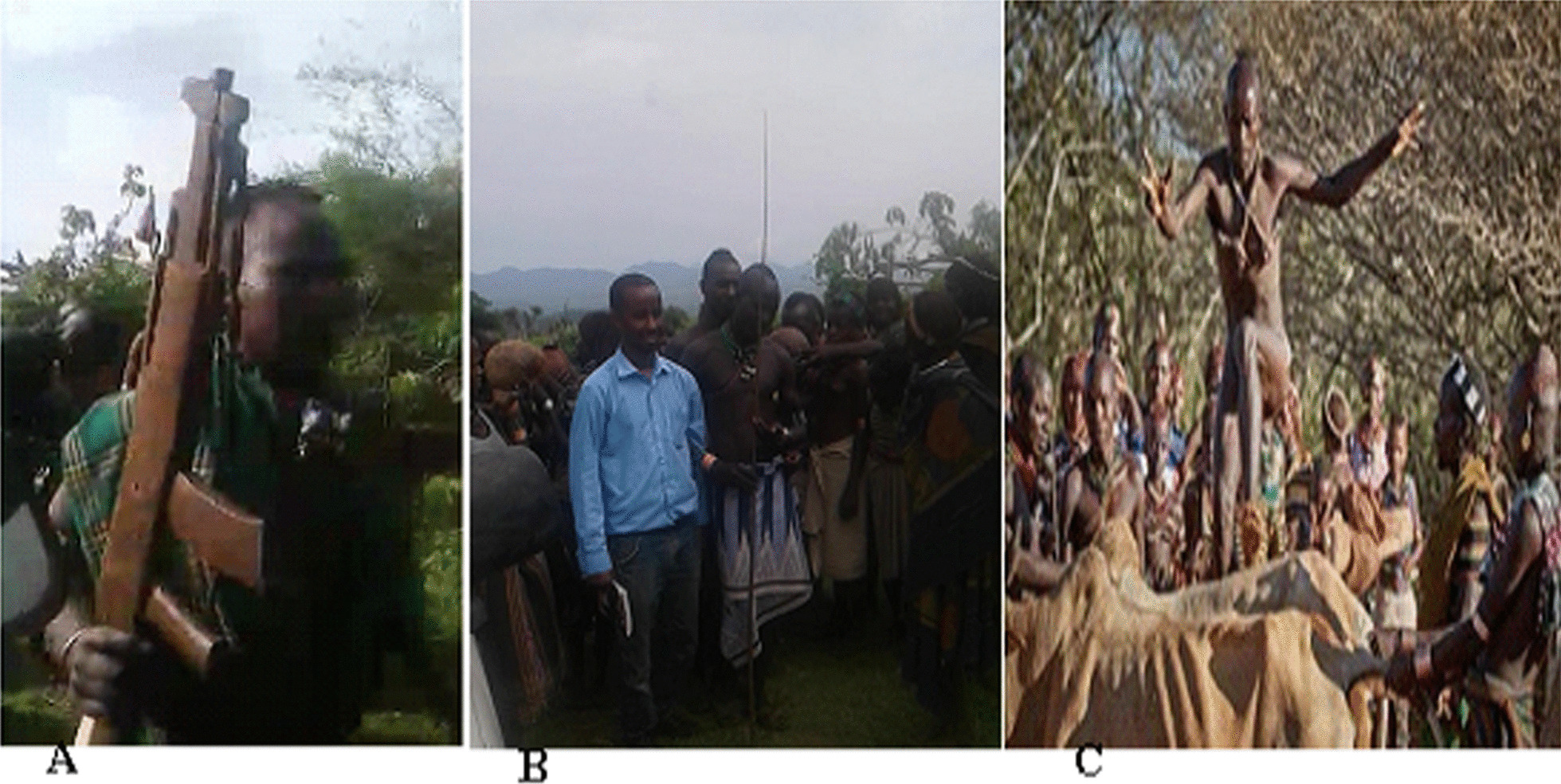
**A** ***ivangadi*** dancing, **B** being accompanied for the bull jumping and **C** ***Ukuli-bulla*** (the bull jumping ceremony of Hamar tribes) (photo by Melese Bekele, 20 March 2019)

The Hamar are the most populous ethnic groups in the Omo and Woito Rift Valley Mountain ranges. They are linguistically and culturally related to tribes from the northern mountains such as the Ari, Banna, and Bashada (unpublished report of the agricultural bureau of Hamar district, 2019). The Hamar people are well-known for their unique ***ivangadi*** dancing and bull jumping *(*locally called ***Ukuli-bulla****)* ceremony (Fig. 2).

**Fig. 3:**
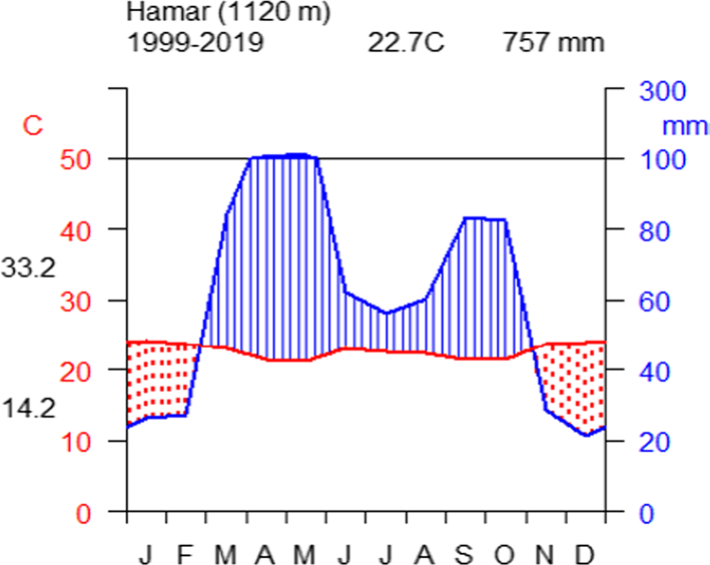
Climate diagram of the study area from 1999 to 2019

About 95% of the district’s coverage has arid and semi-arid climatic conditions, with highly variable mean annual precipitation of 757 mm and an average annual temperature of 22.7 °C. The district has a bimodal rainfall pattern, with the main rainy season occurring between March and May and a shorter rainy season occurring between September and October (Fig. 3). The district experiences a longer dry season from the beginning of November to the end of February. Whereas the average monthly maximum temperature of the hottest month and the average monthly minimum temperature were 33.2 °C and 14.2 °C, respectively (Fig. 3).

### Ethical consideration

The Department of Plant Biology and Biodiversity Management (Addis Ababa University) wrote the official letter outlining the purpose of the study. As a result, the South Omo Zonal and the Hamar district officials were notified, and they forwarded information about the study's objectives to the community leader (***Bitto***), requesting his consent. The community leader has had a thoughtful discussion with the investigator about the study's intentions. The information was then relayed to the respective local leaders by the community leader in order to obtain permission. They scheduled the investigator to return in a week for the next trip, giving them enough time to discuss and decide whether or not to carry out the action.

**Fig. 4:**
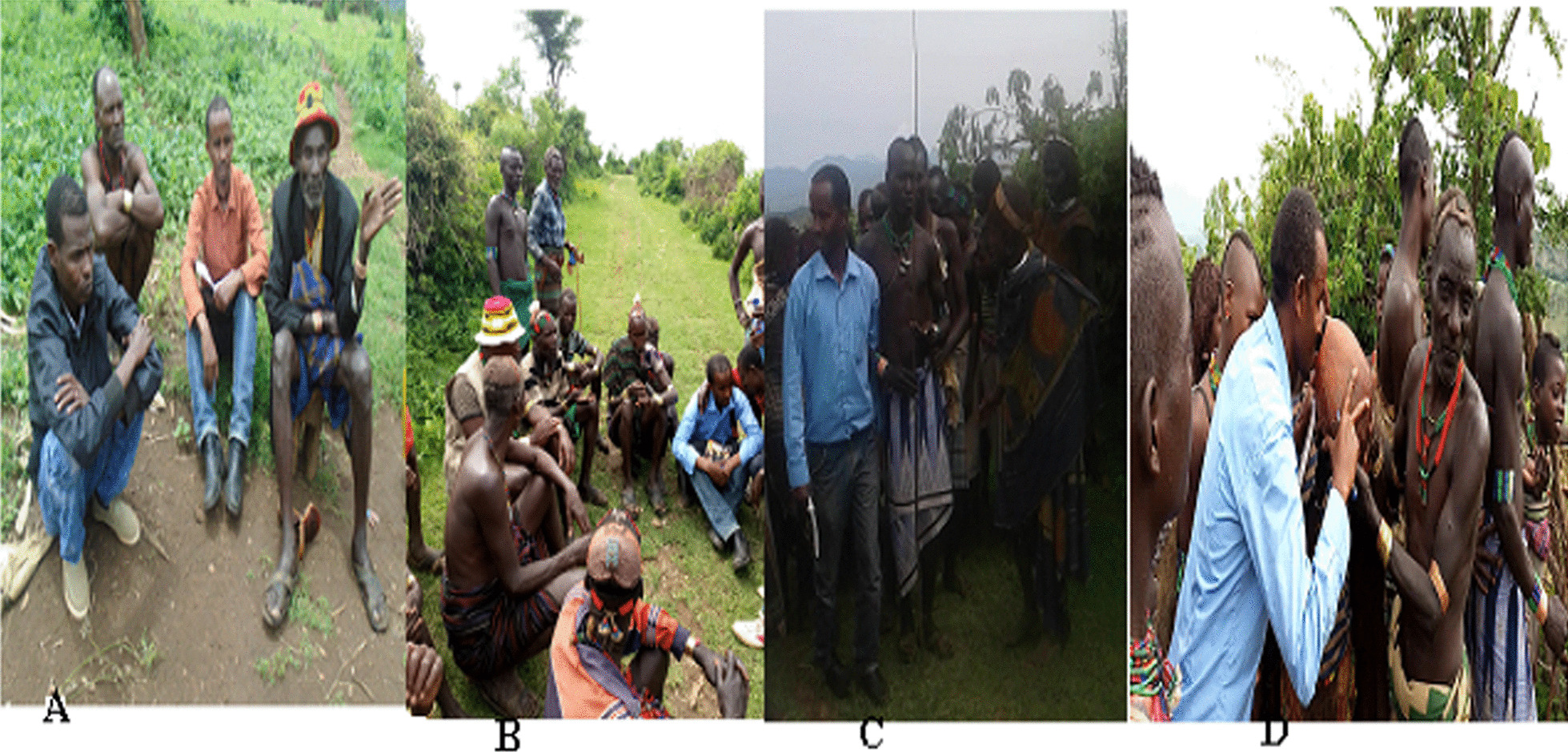
Discussion with a community leader (**A**), with respective local leaders (**B**), traditional song (**C**) and being invited ‘*Farsi’* (**D**) (Photo by Melese Bekele, 15 December 2018)

A week later, the investigator returned to the research site and held a detailed discussion on the purpose of the study, explaining the significant reasons for data collection with the assistance of a native translator (*Hamar Apho* journalist), and finally, verbal consent was obtained (Fig. 4). Then and there, they recognized the investigator as their intimate, locally known as “***Misso***,” meaning “friend,” by the blessings of the elders (***Donza***) and inviting the interesting traditional music of the community's youngsters as well as a lovely cultural beverage of the community called ‘***Farsi***,’ which was made of sorghum(Fig. 4).

### Site selection

A reconnaissance inspection was conducted from 10–29 November 2018 to gain a general understanding of the biophysical and accessibility of the mountain range vegetation, interaction with surrounding communities, and indigenous knowledge-practice-belief systems in the area.

Based on scouting data, the twelve (12) lowest administrative units (locally known as Kebeles/***Tsinti*** in the Hamar language) were prioritized and chosen based on their proximity to the vegetation as well as the community's interaction with the natural vegetation. As a result, the Kebles, namely Gedbak, Lala, Zegerma, Bonkole lagi, Bonkole cherkeka, Kufar, Achikunbulti, Shanko, Dimeka Zuriya, Wero, Dega Keja, and Erbore, were purposefully chosen for the study.

### Informant selection

A total of 326 informants were selected using Cochran’s (1977) formula as:

$$n = \frac{N}{{1 + N\left( e \right)^{2} }}$$

where *n* = sample size; *N* = total number of households in sample villages/Kebeles (1748); *e* = maximum variability or margin of error 5% (0.05) and 1 = the probability of event occurring.

Based on [18], stratified random sampling was used to select general informants and purposive sampling approaches were used to select key informants. Background information of the respondents, including (age, gender, ethnicity, language, religion, and occupation); experience with medicinal plant use, health problems encountered, diagnosis and treatment, the local name of medicinal plants used, growth form, part used, methods of preparation and application, threats to medicinal plants, and conservation practices were meticulously recorded. The age of informants in the study ranges from 20 to 88 years old and classified in to three categories: young (20–35) years old, middle (36–65) years old, and elders (66 and above) years old. Out of the total informants, 74 (48 males and 26 females) were key informants chosen from all Kebeles purposively with the help of the Kebele administrators, personnel in the Agriculture and Rural Development Office of the study Kebeles and peer- recommendations of the local community leaders based on [18]. Interviews and discussions were conducted in the Hamar Language (Hamar Apho), as it is most popularly spoken language in all the study villages (Table 1).

**Table 1:** Number of households and informants in each Kebele

Kebeles	No HH	Total informants	General informants	Key informants	Gender	
					Male	Female
Gedbak	131	25	19	6	19	8
Zegerma	127	24	18	6	19	8
Kufar	143	27	21	6	19	8
Lala	164	30	23	7	19	9
Bonkolelagi	138	26	20	6	19	8
Bonkolecherkeka	156	29	23	6	19	8
Achikunbulti	132	25	19	6	19	8
Shanko	149	28	22	6	19	8
Dimeka zuriya	171	31	24	7	19	9
Wero	153	28	22	6	19	8
Degakeja	144	27	21	6	19	8
Erbore	140	26	20	6	19	8
	1748	326	252	74	228	98

The interviewed number of informants from each Kebele was based on the proportion of households in each Kebele by using the formula:

$${\text{No of informants from each Kebele}} = \frac{{{\text{No of households in each Kebele}} \times {\text{total No of informants}} }}{{\text{ Total No of households}}}$$

### Data collection

Following [18, 19], six different field visits within (March 1–30, 2018; December 1–30, 2018; April 1- 30, 2019; August 1–30, 2019; February 1–30, 2020; and December 1–30, 2020) were conducted to collect ethnobotanical data. During the collection, different seasons and yearly rainfall status were taken in to consideration as factors that can affect the vegetation over the course of the field visits. Semi-structured interviews, detailed discussion with knowledgeable informants, focus group discussions, a guided field walk, and a market survey were all carried out to collect the required ethnobotanical data and the associated indigenous knowledge as well as the management status together with major conservation threats in the area.

**Fig. 5:**
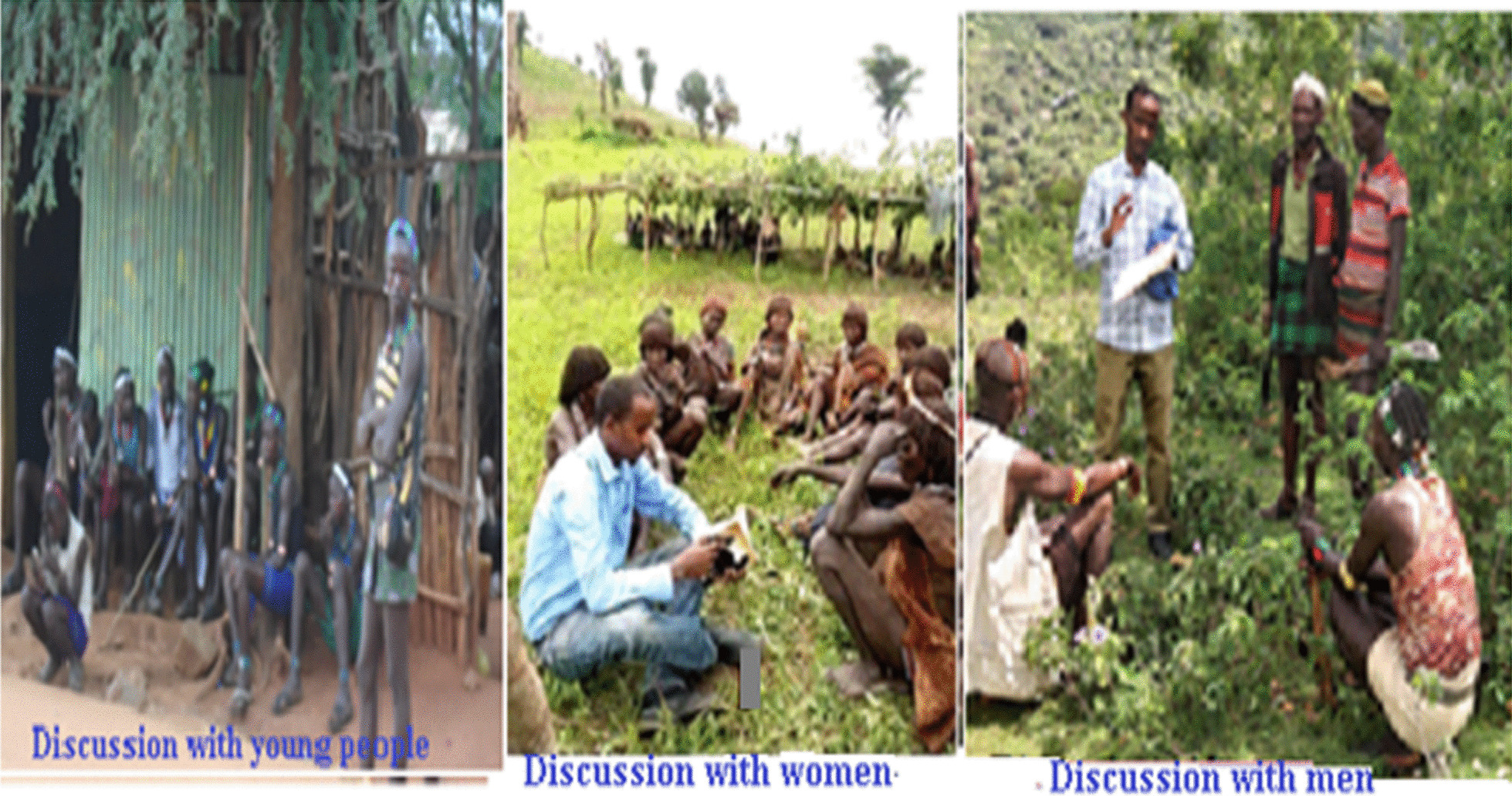
Focus group discussions (Photo by Melese Bekele, 10–20 August 2019)

Twelve independent focus group discussions were held following Martin's [18] to gain additional information on plant use knowledge and to validate the data collected through semi-structured interviews. As a result, each group had 12 members (six from each Kebele involving four males and 2 women) (Fig. 5).

**Fig. 6:**
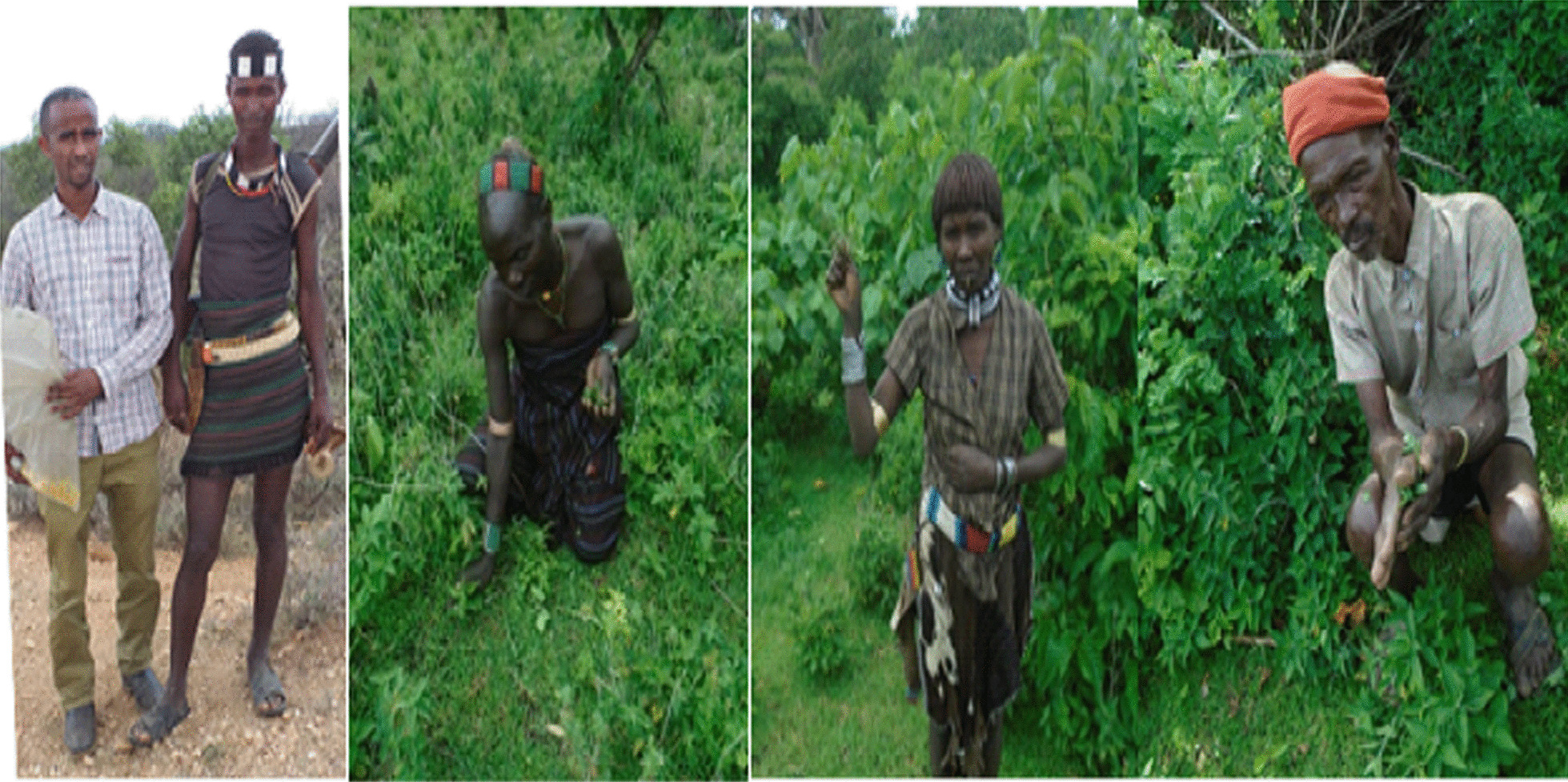
The key informants explaining the source, parts used and methods of preparation of traditional medicines in guided field walk (Photo by Melese Bekele 10, February 2020)

Guided field walks were conducted in assistance with a field guide, who knows the local culture and language/*Hamar Apho* journalist*/*from the South Omo Zone and three key informants from each Kebele in order to obtain essential ethnobotanical explanations about the plant species as well as to gather plant specimens by recording all the necessary information of the particular plant species (Fig. 6).

**Fig. 7:**
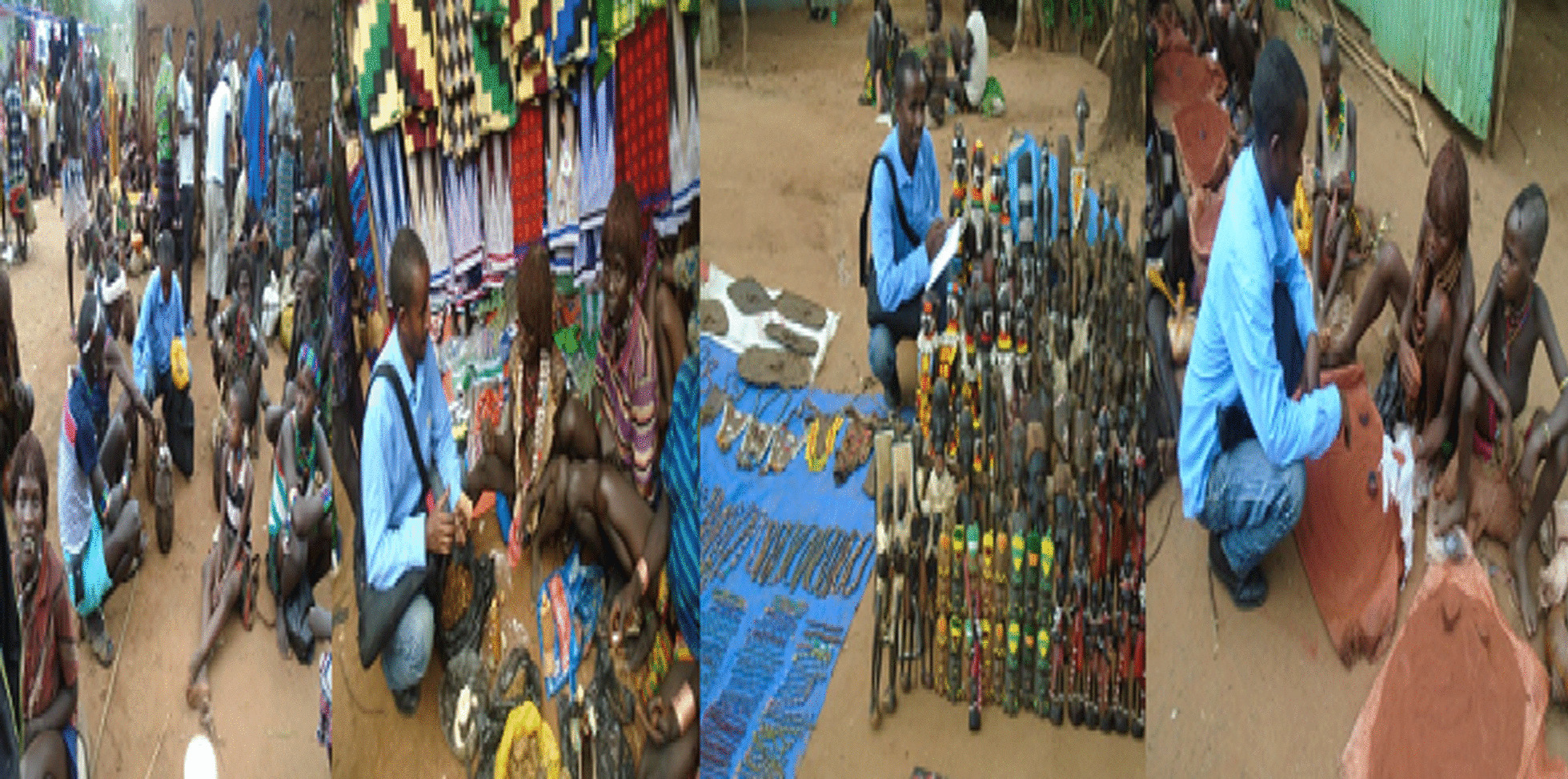
Verbal interviews being conducted with open market sellers (Photo by Melese Bekele, within market visits from March 2018 to December 2020)

Similarly, six market observations were made following [19]; in each field visit, interviews were conducted with open market sellers and buyers of plants and plant products to collect qualitative and quantitative data (Fig. 7).

### Specimen identification

The collected specimens were properly pressed, numbered, dried, and transported to Addis Ababa University, Ethiopian National Herbarium (ENH) for further taxonomic identification to species level using the Flora of Ethiopia and Eritrea, as well as by comparison with the preserved authentic specimens in the herbarium. Then the identified specimens were checked and confirmed by the research Supervisor (Professor Sebsebe Demissew/the Professor of Plant Systematics and Biodiversity). Moreover, the secured voucher specimens with all necessary information were deposited at the herbarium.

### Analysis of the data

The composed data were summarized and organized based on [18] in such a way that can be articulated in terms of percentages and proportions computed during analysis. The relative local and cultural importance of the species cited by the informants for different major use categories besides the medicinal uses were also prioritized and quantified following [20, 21]. Both quantitative and qualitative data analysis methods were employed for preference ranking, fidelity level index (FL), informant consensus factor (ICF), paired comparisons. Jaccard’s coefficient of similarity, and direct matrix ranking applied following [18, 22] to compute and evaluate the levels of importance of one plant species over the other. Statistical Package of Social Sciences (SPSS) version 20 (Spss, I., 2011), was used to display ANOVA of correlation model for ethnobotanical knowledge and age categories. The Jaccard’s coefficient of similarity was computed to comprehend the degree of species similarity with various other related studies conducted in Ethiopia.

### Preference ranking

Following [18], a preference ranking was computed for the most commonly reported medicinal and other use categories of the community. As a result, the preference ranking of eight plant species used against the area's most commonly reported cattle disease and the most prevalent human disease were determined. Similarly, the preference of ten plant species used for local house construction, seven plant species for timber production, six plant species for wild food, eight plant species for forage/fodder, six plant species for shade, and six plant species for honey production were ranked by the number of purposefully selected key informants. This helps to indicate the rank order of the most preferred plants used by the community for the evaluated use.

### Direct matrix ranking

For eight multipurpose plant species chosen from medicinal plant species data, a direct matrix ranking was performed based on the evidence gathered from informants. Eight purposely selected key informants were enquired to rate eight different types of uses, including fodder, food, firewood, construction, bee forage, furniture, shade and charcoal production, assigning use values of each attribute (5 = best, 4 = very good, 3 = good, 2 = less, 1 = least used, 0 = not used). Based on data obtained from informants, the average value of use diversity for each species was taken and finally, the values of each species were summed up and ranked.

### Paired comparison

**Fig. 8:**
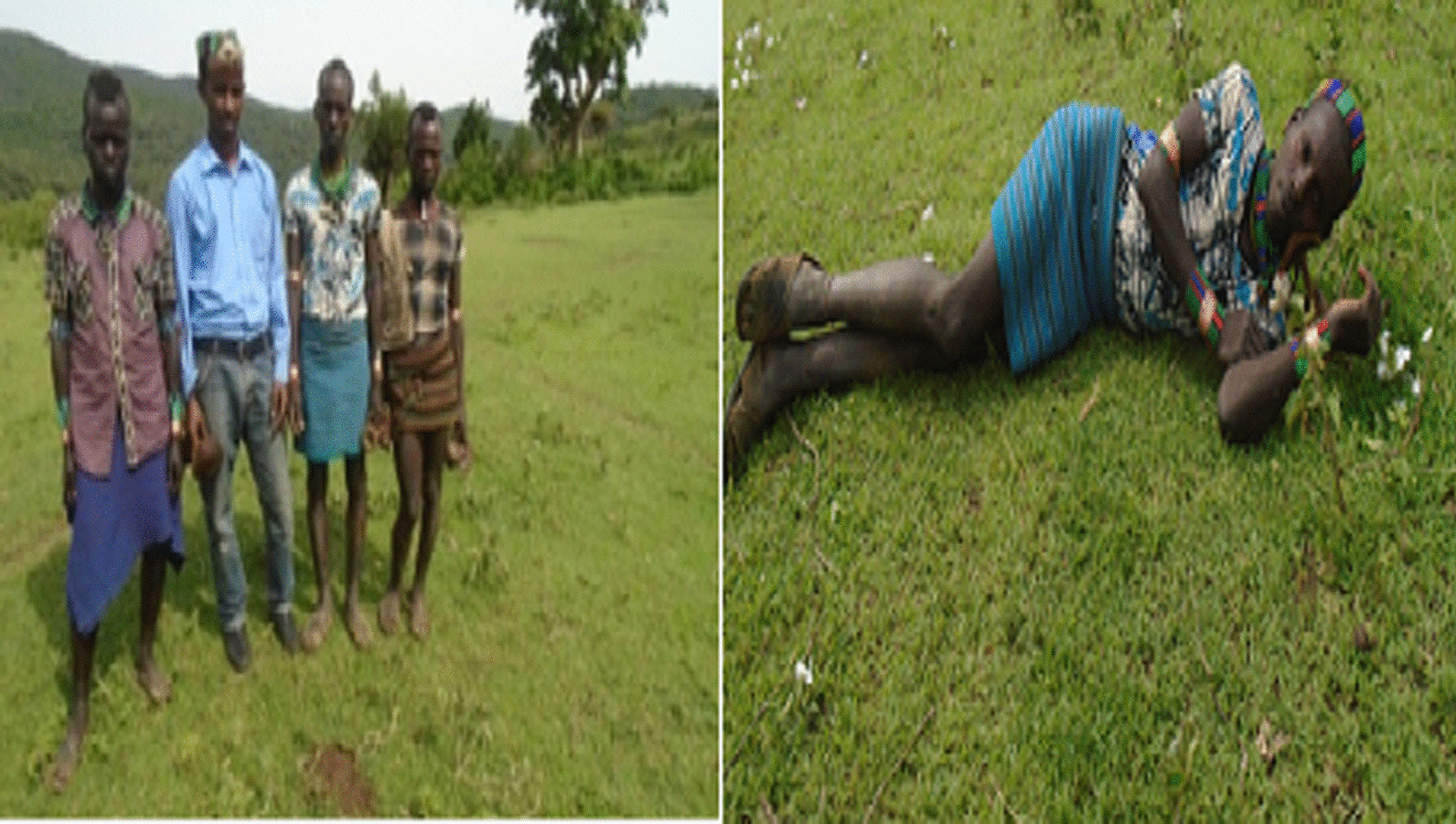
Showing the use of ***Borkota*** (Photo by Melese Bekele, 10 December 2020)

Paired comparison analyses were performed on traditionally important medicinal plant species for treating the area's most common problem of humans and livestock. The other was on the plant species used to make the most popular tool known as Borkota, which is usually found in the hands of Hamar males and is used to seat whenever necessary or to get rest lying supported their neck and head by it during tired walking a long distance on foot (Fig. 8).

Accordingly, eight key informants were selected and responded independently for seven traditionally important plants species selected for treating the snake poison and the values were summarized. Similarly, five highly cited plant species were evaluated by eight key informants to indicate the community’s preference of plant species used to make *Borkota* in the area.

### Informant consensus factor

The agreement of people across different medicinal plants as well as the most popular plant species used for other purposes was tested by calculating informants consensus factor (ICF) following [23]. Accordingly, the ICF on medicinal uses of different plant species was calculated as: a number of use records in each type (*n*_ur_) minus the used species number (*n*_t_), divided by the amount of use citations in each type minus one [24]. The factor delivers an array of 0 to 1, where a high value considered as an indicator for a high rate of informant consensus.

$${\text{ICF}} = \frac{{n_{{{\text{ur}}}} - n_{{\text{t}}} }}{{n_{{{\text{ur}}}} - 1}}$$

where ICF = Informant Consensus Factor, *n*_ur_ = number of uses citation of each category, *n*_t_ = the used species number.

However, some of the limitations of this method were taken in to consideration as it does not distinguish degrees of importance and analyzes only the average number of cited uses. Consequently, a plant having two listed purposes but rarely used could be considered as more "important" than a very popular plant with only one use.

### Fidelity level index

Fidelity level is useful for identifying the key informants’ most preferred species used for treating certain ailments. The medicinal plants that are widely used by the local people have higher FL values than those that are less popular. Fidelity level shows the percentage of informants claiming the use of a certain plant species for the same major purpose. This is designed to quantify the importance of the species for a given purpose. As a result, fidelity level index was quantified for ten medicinal plant species commonly used to treat the most common problem (rabies) in the area.

$${\text{FL}} = Np/N\quad {\text{or}}\quad {\text{FL}}\% = Np/N \times 100$$

where FL = Fidelity Level and FL%, the percentage fidelity level, *Np* = the informants number who independently suggested the application of one species for similar major purpose, and *N* = the informants number who stated a species for any use.

This index could be articulated in percentage, that value close to 100% (1) showing that almost all the uses mentioned refer to the same purpose (the plants and their use for a particular purpose are most preferred), whereas low FLs are generally obtained for plants that are used for many different purposes. In other words, it is believed that medicinal plants that are frequently used to treat the same type of ailment are more likely to be biologically active. However, due to the complexity of cultural and pharmacological realities, fidelity level may not adequately capture a proxy for effectiveness or even new drug discovery.

### Cultural Significance Index (CSI)

The index of cultural significance calculates the importance of the plant species through the researcher-determined weighted ranking of multiple factors [25]. Then [26] has assigned scores on a five-point scale to the variables of quality and intensity of use and allocated a score of two, one, or 0.5 for the exclusivity or preference of use. However, [25] revised the approach with a two-point scale for the variables to reduce the subjectivity. The revised method also assimilated a consensus technique called a correction factor to decrease the sensitivity of the technique to sampling intensity. The ethnographic, qualitative method of the CSI technique needs considerable experience and understanding with a cultural assembly for meaningful results. The Cultural Significance Index, based on [25] computed as:

$${\text{CSI}} = \mathop \sum \limits_{i = 1}^{n} \left( {i*e*c} \right)*{\text{CF}}$$

where *i* = species management [managed (2) or non-managed (1)], *e* = Use preference [preferred (2) or not preferred (1)], *c* = Use frequency [frequently used (2) or rarely used (1)], CF = Correction factor [the number of informant citations for a given species divided by informant number records for the most cited species]. The subjective allocation methods can save time in the field and provide a more refined dataset than the “uses totaled” method. However, these methods introduce researcher bias because degrees of importance and categories are based solely upon researcher assessment. Furthermore, as with other methods in this category, informant responses are not independently recorded, thus eliminating the opportunity for analysis of informant variability.

### Jaccard coefficients of similarity (JCS)

The Jaccard’s similarity coefficient was computed to determine the species similarity in composition with five other studies done in different part of the country. The JCS was calculated using the formula:

$${\text{JCS}} = \frac{c}{{\left( {a + b + c} \right)}}$$

where JCS is the Jaccard’s Coefficient of similarity, *a* is the number of species in habitat A (in Buska Mountain range), *b* is the number of species in habitat B (in other study areas), and *c* is the number of common species occurring in habitat A, and B. The percentage JCS is obtained by multiplying by 100. Statistical Package of Social Sciences (SPSS) version 20 [27], was used to display analysis of variance (ANOVA) of correlation model for ethnobotanical knowledge and age categories.

## Result

### Agro-ecology and socio-demography of the study area

Pastoralism and semi-pastoralism are the two main land use systems in the study area. Communities residing in relatively lowland areas, where crop cultivation is extremely difficult due to harsh climate conditions, rely on pastoralism. The bulk of the villages we visited as a result, such as Erbore, Degakeja, Wero, Zegerma, Kufar, Gedbak, Bonkolelagi, and Bonkolecherkeka, where there are pastoralist communities that only relied on the mixed livestock products (cattle, goats, and sheep). In contrast, people who live in comparatively highland regions like Lala, Achikunbulti, Shanko, and Dimeka zuriya are semi-pastoralists who equally rely on plant and animal goods. They mostly farm sorghum and maize as their main food crops and a source of cattle products, which they also use. Hamar, Kara, and Erebore are the three main ethnic groups in the study communities, with Hamar receiving the most attention. Most members of the community practice cultural religion (Table 2).

**Table 2:** Agroecology and socio-demography of the study area

Name of the study villages	Altitude range	Socio-demographics			Type of agro-ecology exist
		Population	Ethnicity in %	Religion in %	
Gedbak	1324–1731	1672	Hamar = 70 Kara = 16 Erbore = 9 Other = 5	Christian = 20 Muslim = 3 Others = 77	Mainly low land
Zegerma	982–1521	1622	Hamar = 82 Kara = 11 Erbore = 5 Other = 2	Christian = 18 Muslim = 7 Others = 75	Lowland with some escarpment
Kufar	1126–1845	1822	Hamar = 76 Kara = 7 Erbore = 6 Other = 11	Christian = 14 Muslim = 1 Others = 85	Lowland with some escarpment
Lala	1322–2100	2086	Hamar = 90 Kara = 5 Erbore = 2 Other = 3	Christian = 26 Muslim = 5 Others = 69	Vast majority of the village is highland and mountainous
Bonkolelagi	1141–1760	1760	Hamar = 64 Kara = 19 Erbore = 12 Other = 5	Christian = 14 Muslim = 2 Others = 84	Lowland and cliffy
Bonkolecherkeka	1029–1974	1985	Hamar = 69 Kara = 10 Erbore = 15 Other = 6	Christian = 8 Muslim = 1 Others = 91	Lowland with some escarpment
Achikunbulti	856–1923	1684	Hamar = 66 Kara = 16 Erbore = 14 Other = 4	Christian = 15 Muslim = 2 Others = 83	Relatively highland and mountainous
Shanko	1213–2005	1897	Hamar = 74 Kara = 11 Erbore = 9 Other = 6	Christian = 20 Muslim = 3 Others = 77	Relatively highland
Dimeka zuriya	1120–1867	2174	Hamar = 60 Kara = 8 Erbore = 12 Other = 20	Christian = 35 Muslim = 10 Others = 55	Lowland with some escarpments and is where the district's central town is located
Wero	1006–1685	1948	Hamar = 65 Kara = 18 Erbore = 10 Other = 7	Christian = 12 Muslim = 1 Others = 87	Lowland with some escarpment
Degakeja	1230–1952	1496	Hamar = 68 Kara = 17 Erbore = 13 Other = 2	Christian = 19 Muslim = 3 Others = 88	Lowland with most escarpment coverage
Erbore	380–1654	1468	Hamar = 11 Kara = 14 Erbore = 68 Other = 6	Christian = 22 Muslim = 5 Others = 83	The village in the study with the hottest climate and the lowest altitudinal range

Key: Religion ‘others’ = Cultural, Total population 20,146

### Diversity of medicinal plant species used by the local community in the area

A total of 145 medicinal plant species used to treat human and livestock ailments were collected and documented from the study area. The record of major human and livestock ailments in the area, as well as the number of plant species used by the local people, revealed that 74 (51%) of the traditional medicinal plants were reported to be used only for humans, 42 (29%) were used to treat both human and livestock disorders, while 29 (20%) were used to treat only livestock diseases. The plant families Fabaceae (33 species, 22.8%), Asteraceae (16 species, 11%), and Lamiaceae (14 species, 9.6%) were found to be the most dominant families in the area.

**Fig. 9:**
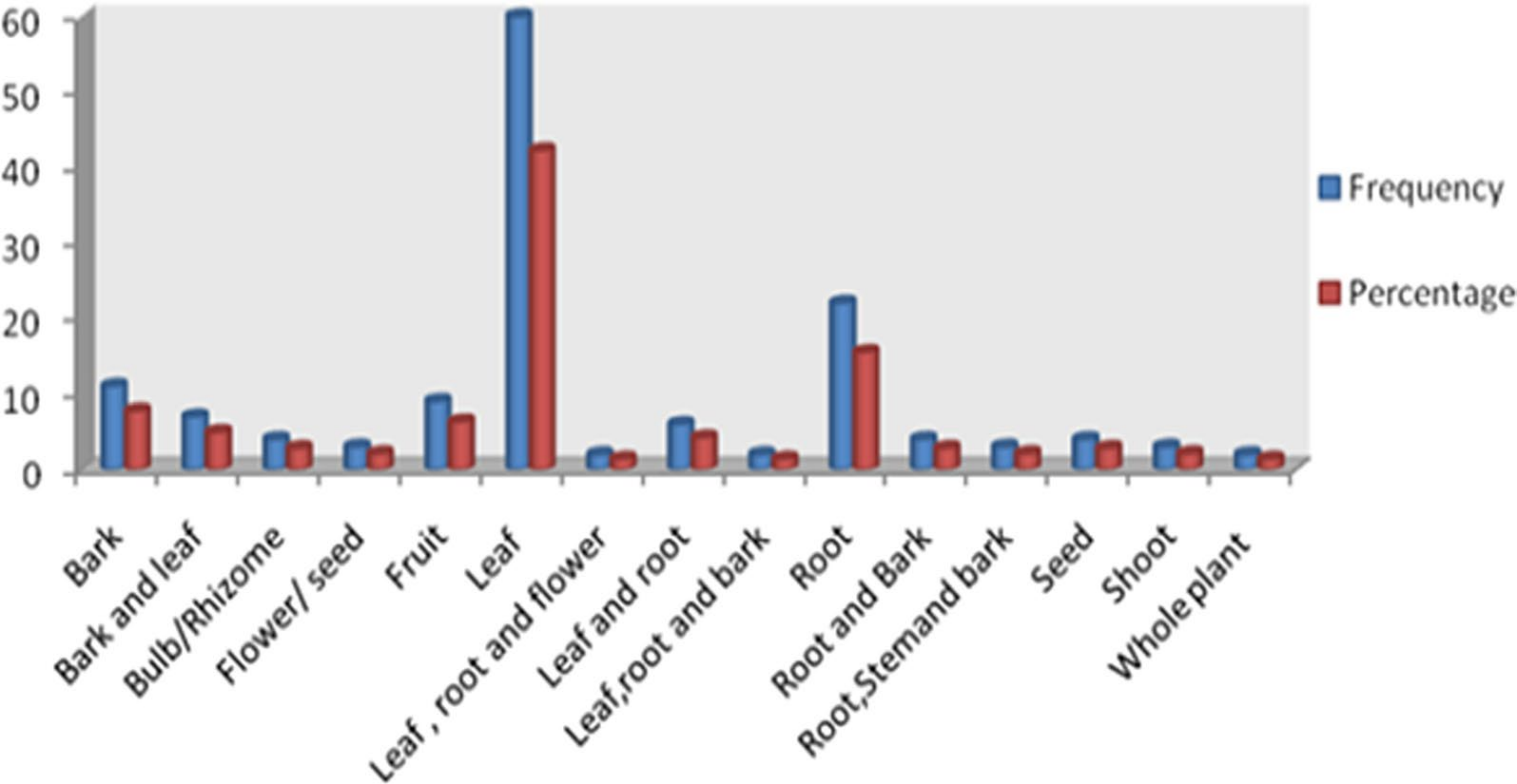
The percentage of medicinal plant parts used for preparing the remedy in Hamar

Majority of the medicinal plant species (72%) were collected and identified from the wild, 21% were from home gardens, and the remaining 7% were recorded as semi-wild, which were found frequently both in the wild and at home gardens. Shrubs contributed the most to the growth forms of traditional medicinal plant species (42.2%), followed by herbs (24.7%), trees (23.9%), herbaceous climbers (6.4%), and lianas/woody climbers (2.8%). The leaf has the highest proportion (42.3%), and is the most commonly used medicinal plant parts used for preparing the remedies, followed by the root, contributing 15.5%, and the bark, which added 7.5% of the parts used (Fig. 9).

About 78.8% of the medicinal plant parts were reported by the informants to be used freshly, 14.1% was at dry status, and the remaining 7.1% could be either in dry or fresh state. The most frequently cited route of application of remedies in the area was oral (57.7%), followed by dermal application (14.8%). Drinking (42%) was the most frequently reported method of administration when recognizing the overall modes of application of the remedies, followed by chewing and swallowing (23%). Crushing (38%) was the most common method of preparation of traditional medicines in the area, followed by squeezing (33%) and pounding (19%). Besides, more than 70 different human and livestock ailments were identified as health issues in the area and are being treated by various plant species. Malaria was reported by 54% of the respondents as the most prevalent health problem in the area, followed by wounds (22%), snake bites (18%), and tapeworm (12%).

### Dosage of medicinal plant remedies and ingredients added

**Fig. 10:**
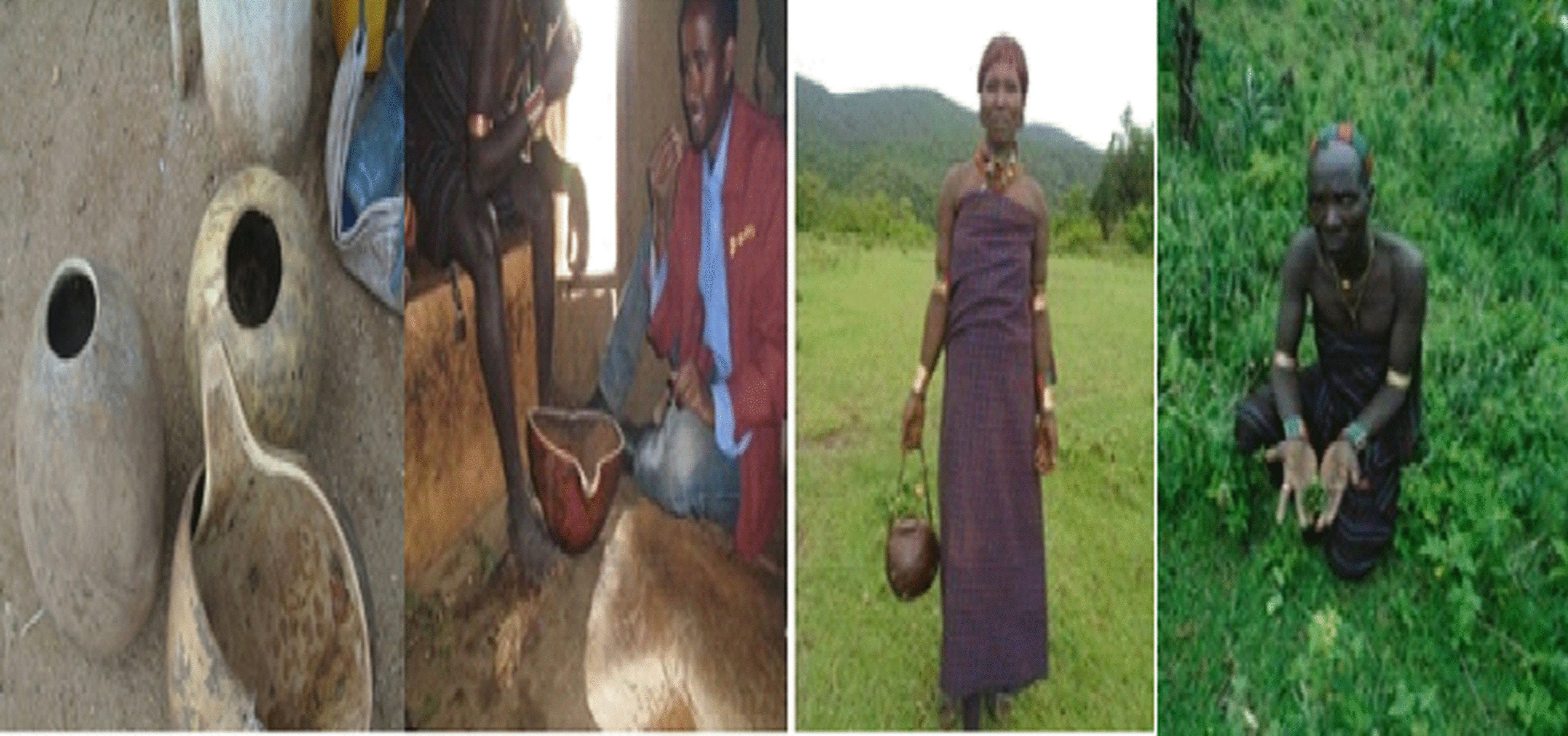
The local materials used to measure the dose of the plant remedies in the study area (Photo by Melese Bekele 15, February 2020)

The communities of the study area have developed knowledge of dosing the remedies based on their historical and long-term practical experience of using traditional medicinal plants for various ailments. Although there were different dosage units and administration periods, the common way of dosage delivery was determined by the degree of severity of the ailments treated, the patient’s or diseased animal’s health status, the age of the sick, and the experience of the local healer who administers the remedy. The informants also mentioned that various additives and solvents, including salt, coffee, tea, honey, milk, butter, soil, and water, are added to when preparing traditional remedies in the area. These ingredients were claimed to be used to reduce side-effects and improve the taste and lower the chances for poisoning. To measure the amount or dose of the remedy, various tools were used, including *Carba*, *Onkolo*, *Basala shorka* [all of which were made of Gusi (*Lagenaria siceraria*) and only differed in the size of the openings], finger length, coffee cup, tea cup, and others were used to measure the amount or dose of the remedy (Fig. 10).

### Efficacy of medicinal plant remedies

The informant consensus factor (ICF) analysis revealed that about 72 human and livestock ailments were considered within eleven disease categories, with the reproductive problem category scoring the highest ICF value (0.94); treated by 5 species and 72 use citations, followed by the category of gastrointestinal disorder, which scored an ICF value of 0.92 and was treated by 8 plant species and 97 use citations, while the lowest ICF value 0.87 was recorded for the category of dermal problems, treated by 7 species and 50 use citations(Table 3).

**Table 3:** Diseases categories with Informant Consensus Factor in the study area

Diseases category	Number of plant species (*N*_t)_	Number of use citation (*N*_ur_)	ICF
Malaria, fever, Sudden sickness, discomfort and headache	10	82	0.88
Gastrointestinal disorder(dry stomach, abdominal problem and diarrhea)	8	97	0.92
Allergic reaction, ecto-parasites, itching & skin ulcer	9	82	0.90
Snake bite, rabies and harmful insects bite	6	63	0.91
Evil spirit, epilepsy and evil eye	8	60	0.88
Tapeworm, ringworm, leeches and ascaris	7	56	0.89
Respiration problem and tonsillitis	5	41	0.90
Tuberculosis, common cold, blackleg/anthrax, & Pneumonia	8	82	0.91
Reproductive problem (Placental retention, menstrual problem, gonorrhea)	5	72	0.94
Dental (Tooth ache, teeth brushing and gum bleeding)	7	50	0.87
Hemorrhoid, wound and swelling	6	62	0.91

The level of agreement among informants on each medical plant and the propensity of some medicinal plants to treat particular health issues were carefully evaluated. Accordingly, *Phytolacca dodecandra* had the highest fidelity level (94.1%) for treating rabies, followed by *Albizia anthelmintica* with a fidelity level of 88.3% for treating tapeworm and *Moringa stenopetala* with a fidelity level of 83.3% and used to treat cold and coughs in the area (Table 4).

**Table 4:** Shows the fidelity level of the medicinal plant species commonly used to treat the most common human diseases in the area

No.	Medicinal plants	Disease type	*Np*	*N*	FL%
1	*Albizia anthelmintica*	Tapeworm	8	9	88.9
2	*Allium sativum*	Tuberculosis	6	11	54.5
3	*Capparis tomentosa*	Evil eye	4	7	57.1
4	*Cordia sinensis*	Respiratory problem	3	5	60
5	*Croton macrostachyus*	Muscle pain	4	9	44.4
6	*Gardenia ternifolia*	Malaria	3	7	42.8
7	*Lannea fruticosa*	Wound or swelling	5	8	62.5
8	*Moringa stenopetala*	Cold and cough	5	6	83.3
9	*Phytolacca dodecandra*	Rabies	16	17	94.1
10	*Solanum incanum*	Snake bite	6	8	75

Where *FL* fidelity level, *Np* the number of informants who suggested the use of a species for the same major purpose, and *N* the informants number who mentioned a plant for any use

On the other hand, about 20% of the traditional medicinal plant species collected and identified from the study area were used to treat only livestock health problems. Furthermore, 29.6% of the plant species recorded for medicinal value was reported to be used to treat human and livestock ailments. The preference ranking of eight plant species used against the most commonly reported cattle disease (diarrhea) in the area revealed that *Protea gaguedi* stands the first, with a preference score of 71, followed by *Amaranthus hybridus* scoring 64 for its significance in the study area (Table 5).

**Table 5:** Preference ranking of commonly used plant species to treat diarrhea of the livestock in the community

Species	K1	K2	K3	K4	K5	K6	K7	K8	K9	K10	Total	Rank
*Adenium obsum*	5	8	4	8	4	8	4	5	7	5	58	3rd
*Amaranthus hybridus*	7	8	5	7	5	7	5	6	6	8	64	2nd
*Clutia lanceolata*	3	2	7	3	2	1	8	7	8	7	48	5th
*Combretum aculeatum*	2	5	5	4	3	3	1	4	5	1	33	7th
*Croton macrostachyus*	6	6	2	5	6	4	3	6	3	4	45	5th
*Galiniera saxifraga*	1	6	7	2	3	2	2	5	1	2	31	8th
*Protea gaguedi*	8	7	8	6	8	6	7	8	7	6	71	1st
*Rhamnus prinoides*	6	7	8	6	7	5	6	1	2	3	50	4th

### Paired comparison

A paired comparison of medicinal plants for humans and livestock for treating snake bite poison (*Guni Dhesha*), which was reported to be one of the most common problems in the study area, was computed. Thus, seven plant species cited for treating snake bite poison were chosen, and eight key informants have assigned values to indicate the effectiveness and status of the plant species. *Solanum incanum*, *Phytolacca dodecandra*, *Securidaca longepedunculata*, and *Stephania abyssinica* were ranked first to fourth respectively. *Verbena officinalis* and *Dombeya torrida* were less preferred, and *Pennisetum setaceum* was the least preferred and least effective when compared to other species (Table 6).

**Table 6:** Paired comparison of seven plant species used to treat snake poison in the area

Plant species	Key informants								Total	Rank
	K1	K2	K3	K4	K5	K6	K7	K8		
*Dombeya torrida*	4	3	3	4	1	2	1	3	21	6th
*Securidaca longepedunculata*	1	7	4	7	2	4	6	5	36	3rd
*Pennisetum setaceum*	3	2	2	1	4	3	2	1	18	7th
*Verbena officinalis*	2	4	5	2	5	1	5	2	26	5th
*Solanum incanum*	7	6	6	5	7	6	5	7	49	1st
*Phytolacca dodecandra*	6	5	7	6	3	5	7	4	43	2nd
*Stephania abyssinica*	5	1	3	3	6	7	3	6	34	4th

### Other uses of the medicinal plant species in the area

The analysis of the medicinal plant species for their multipurpose usage showed that 10% of them were recorded for their food values, 8.9% for local house construction, 11% for fire wood and charcoal production, 4.8% for timber production, 7.6% for farm implementation materials, 9.6% for fodder, 10% for bee forage, 8.3% for shade/livestock shelter and 5.5% for soil fertility/conservation and 2.8% for cultural and ritual values.

### Direct matrix ranking

*Acacia mellifera* took first place in the direct matrix ranking score of ten multipurpose plant species for seven different types of uses besides the medicinal use such as (fodder, food, firewood, construction, bee forage, furniture, shade and charcoal production), followed by *Olea europaea* subsp.cuspidata. In terms of use category evaluation, the use of these plant species for shade received the highest score compared to the other use categories (Table 7).

**Table 7:** Average value of direct matrix ranking of plant species based on their general use values (fod = fodder, foo = food, firew = firewood, con = construction, fora = bee forage, furn = furniture, shad = shade and char = charcoal production)

Plant species	Use categories									
	Fod	Foo	Firw	Con	Fora	Furn	Shad	Char	Total	Rank
*Acacia mellifera*	5	0	5	4	5	4	4	5	32	1st
*Balanites aegyptiaca*	5	5	3	3	4	2	4	1	27	4th
*Cordia africana*	3	2	4	4	3	4	4	0	24	6th
*Olea europaea*	5	1	4	5	4	5	5	2	31	2nd
*Syzygium guineense*	3	4	3	3	4	3	5	1	26	5th
*Tamarindus indica*	3	5	2	3	5	4	5	2	29	3rd
*Terminalia brownii*	4	0	3	4	3	4	3	2	23	7th
*Ximenia americana*	3	5	2	3	4	2	3	0	22	8th
Total	31	22	26	29	32	28	33	13		
Rank	3rd	7th	6th	4th	2nd	5th	1st	8th		

The preference ranking of ten plant species used for local house construction was tested against eight key informants giving the highest value 10 for the most important species and value 1 for the least preferred species for the mentioned purpose. Accordingly, *Acacia mellifera* was the most preferred species in the area for local house construction, followed by *Terminalia brownii* to be the second most important species preferred by the respondents. On the other hand, *Combretum aculeatum* was seen to score the least preference value relative to other species for the local house construction (Table 8).

**Table 8:** Preference ranking of ten plant species used for local house construction in the area

Scientific name	K1	K2	K3	K4	K5	K6	K7	K8	Total	Rank
*Ozoroa insignis*	5	8	9	5	2	4	7	2	42	7th
*Combretum aculeatum*	4	5	6	5	4	1	4	1	31	10th
*Grewia bicolor*	6	7	8	6	1	3	8	5	44	6th
*Celtis africana*	3	10	3	1	9	7	10	6	49	4th
*Teclea nobilis*	7	6	5	7	6	10	6	4	51	3rd
*Combretum adenogonium*	9	1	4	3	3	2	9	3	34	9th
*Acacia mellifera*	10	9	10	9	10	9	5	9	71	1st
*Terminalia brownii*	8	3	5	10	8	8	3	10	55	2nd
*Albizia anthelmintica*	2	4	4	8	7	5	8	7	45	5th
*Acacia seyal*	1	6	7	4	5	6	2	8	39	8th

Timber production was viewed as one of the district's income-generating practices, particularly in the Dimeka town area, but less so in other areas. Because of their practical preference for strength and quality, the plant species used for this purpose are very selective and few in number. The preference ranking of the seven plant species with the highest citation revealed for the purpose during free listing was computed using ten key informants, yielding the highest value of seven (7) for the most preferred species and the lowest value one (1) for the least preferred species. Accordingly, *Cordia africana* was ranked at first, scoring 52 followed by *Juniperus procera* with 46 (Table 9).

**Table 9:** Preference ranking of seven plant species used for timber production in the area

Scientific name	K1	K2	K3	K4	K5	K6	K7	K8	Total	Rank
*Juniperus procera*	5	6	6	7	5	4	4	7	45	2nd
*Prunus africana*	3	2	5	4	3	6	2	5	30	5th
*Millettia ferruginea*	4	1	4	3	5	1	3	4	25	7th
*Podocarpus falcatus*	2	4	4	3	2	3	7	2	27	6th
*Cordia africana*	7	5	7	6	7	5	6	6	49	1st
*Albizia anthelmintica*	1	3	5	4	6	5	5	3	32	4th
*Ficus vasta*	6	7	5	3	4	7	4	6	42	3rd

Similarly, based on the preference of medicinal plant species reported for edibility by the community, the result indicated that *Ximenia Americana* stands at first place for its wild food value followed by *Balanites aegyptiaca* (Table 10).

**Table 10:** The preference ranking of the six most commonly used wild food plant species

Scientific name	K1	K2	K3	K4	K5	K6	K7	Total	Rank
*Balanites aegyptiaca*	1	6	6	6	6	4	5	34	2nd
*Carissa spinarium*	3	1	5	3	4	5	1	22	5th
*Syzygiumguineense*	4	4	3	3	3	3	6	26	4rd
*Tamarindus indica*	5	3	6	5	4	3	4	30	3rd
*Ximenia americana*	6	5	5	6	5	6	6	39	1st
*Vitex doniana*	2	2	4	4	1	2	4	19	6th

The most popular tree/shrub species used for forage/fodder in the area include: *Sesbania sesban, Vernonia amygdalina, Balanites aegyptiaca, Balanites rotundifolia, Maerua angolensis, Terminalia brownii, Acacia mellifera, Dichrostachys cinerea, Piliostigma thonningii, Erythrina brucei, Indigofera hirsuta, Dodonea angustifolia, Sida rhombifolia* and *Gardenia ternifolia*. Accordingly, the preference ranking of eight forage/fodder plant species that had higher citation during free listing was computed using eight key informants scoring the highest value eight (8) for the most preferred species and the lowest value 1 for least preferred one. Thus, *Sesbania sesban* stands at first place followed by *Gardenia ternifolia and Piliostigma thonningii* as the second and third most preferred species respectively (Table 11).

**Table 11:** Preference ranking of eight plant species used for forage/fodder in the area

Scientific name	K1	K2	K3	K4	K5	K6	K7	K8	Total	Rank
*Balanites aegyptiaca*	5	2	4	6	4	8	3	2	37	4th
*Vernonia amygdalina*	3	6	3	2	5	6	2	4	35	6th
*Dichrostachys cinerea*	2	1	2	1	2	4	1	3	18	8th
*Piliostigma thonningii*	8	4	5	5	6	6	7	8	55	3rd
*Gardenia ternifolia*	7	5	7	8	7	2	8	7	59	2nd
*Acacia mellifera*	1	3	1	4	1	5	5	1	26	7th
*Sesbania sesban*	6	8	8	7	8	7	4	6	61	1st
Maerua angolensis	4	7	6	3	3	1	6	5	36	5th

**Fig. 11:**
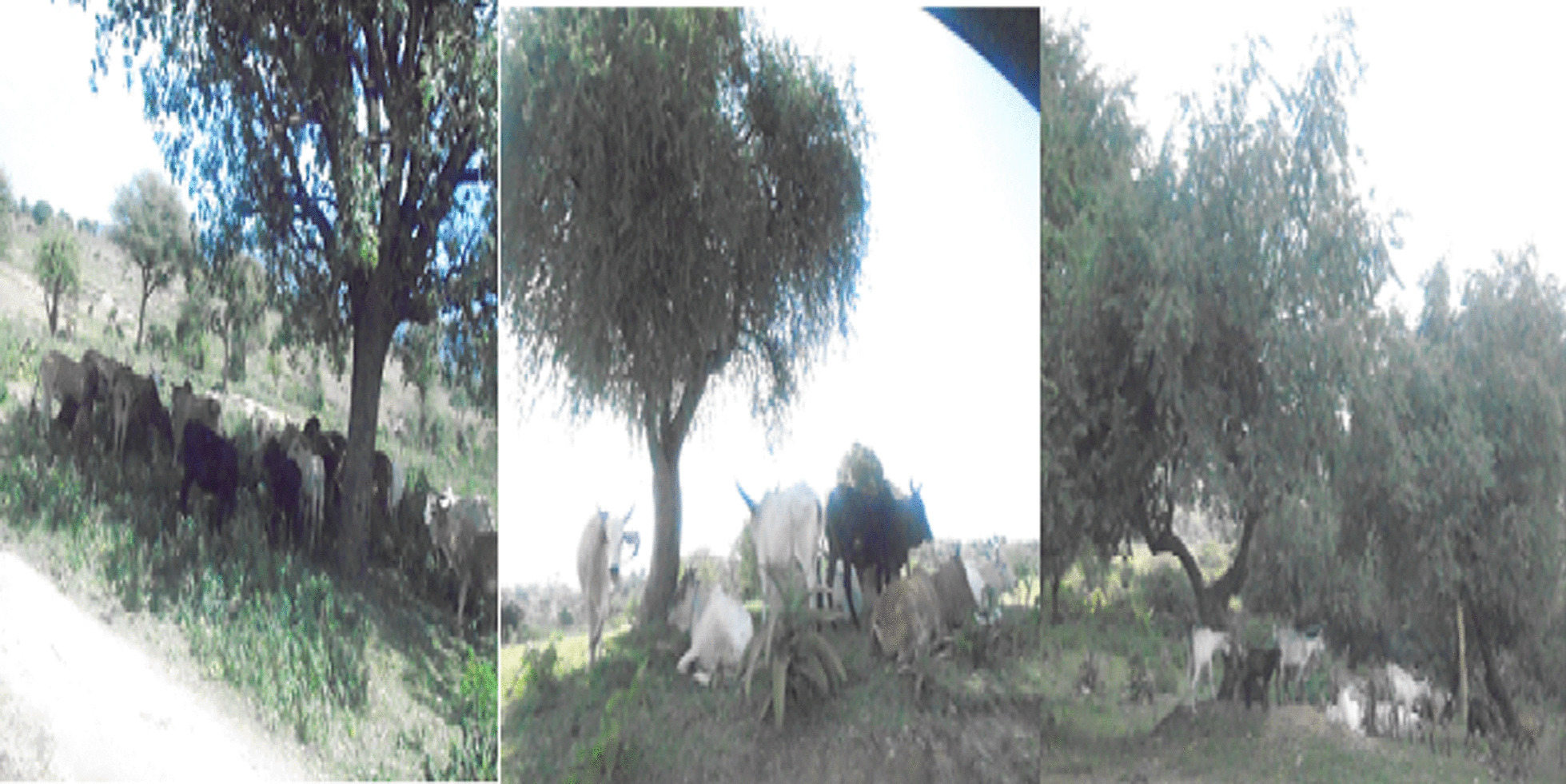
Plant species preferred for livestock shading in Hamar (Photo by Melese Bekele, 2019)

The preference of multipurpose plant species by the local community was also practical for livestock shade, bee foraging/honey production and beehive hanging. It was too treasured that the animals of the community were also able to prefer plant species based on their canopy cover for shading (Fig. 11).

The preference ranking of six plant species used for shade that had higher citations during free listing of species used for shade and ranked by eight key informants indicated that *Tamarindus indica* became the first followed by *Balanites aegyptiaca* and *Celtis africana* as the second and third most preferred species for shade in the area (Fig. 11 and Table 12).

**Table 12:** Preference ranking of six plant species used for shade in the study area

Scientific name	K1	K2	K3	K4	K5	K6	K7	K8	Total	Rank
*Combretum molle*	5	3	4	3	4	1	2	4	26	4th
*Piliostigma thonningii*	3	4	2	4	5	2	3	1	24	6th
*Celtis africana*	1	5	5	5	4	6	3	3	32	3rd
*Balanites aegyptiaca*	2	4	5	6	5	4	5	6	37	2nd
*Ficus vasta*	4	2	3	3	3	3	5	2	25	5th
*Tamarindus indica*	6	7	6	7	6	6	6	6	50	1st

**Fig. 12:**
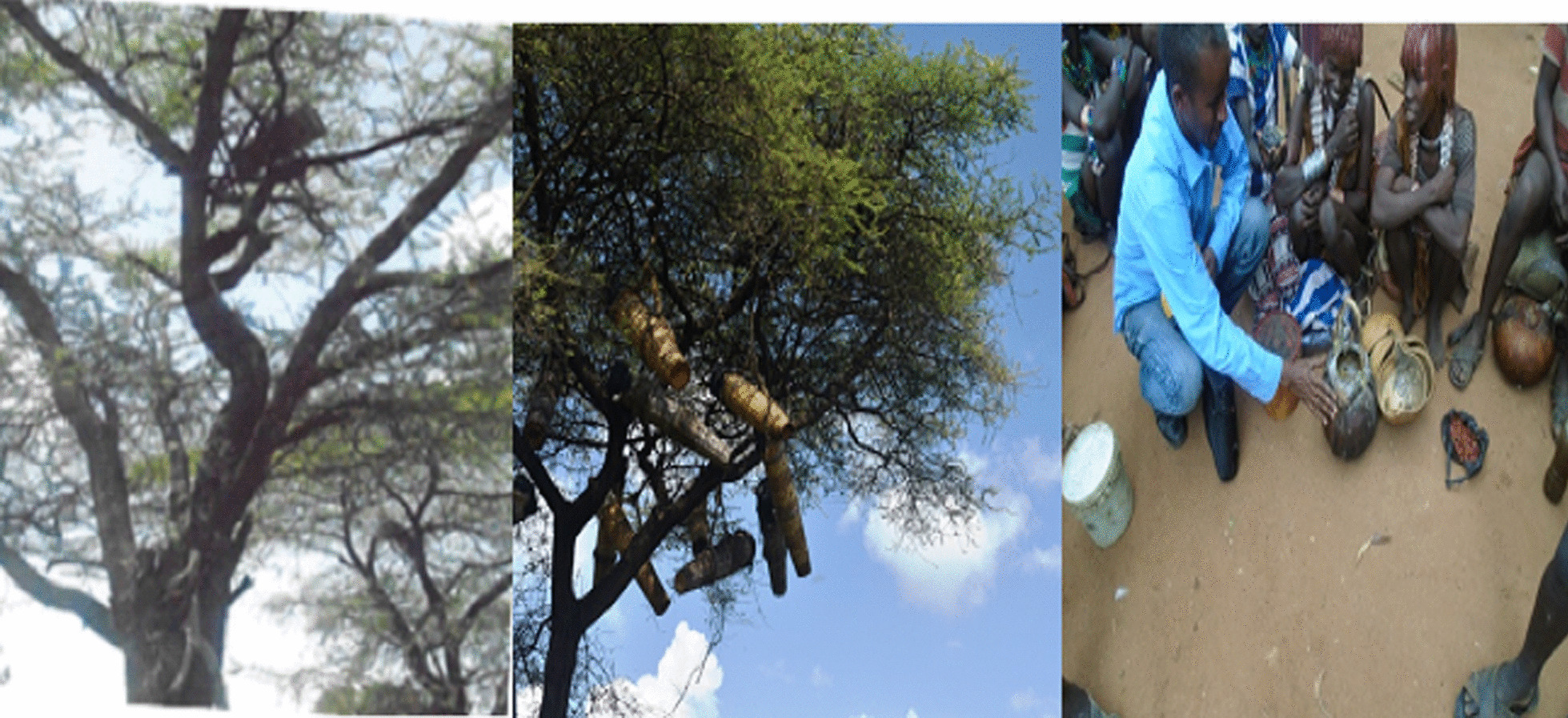
*Acacia tortilis* preferred for bee foraging and traditional beehive hanging in Hamar (Photo by Melese Bekele, 2019)

Correspondingly**,** it was also observed that bee foraging and beehive hanging use categories require own specific species depending on flower characteristics, quality of the honey produced and height of the plant species (Fig. 12).

To evaluate the most favorite plant species for beehive hanging and honey production in the area, preferences ranking of six plant species with higher citation were selected and valued by seven key informants scoring value 6 for highly preferred species and the lowest value 1 for the least chosen species (Table13).

**Table 13:** Preference ranking of six plant species for traditional honey production in the study area

Scientific name	K1	K2	K3	K4	K5	K6	K7	Total	Rank
*Croton macrostachyus*	3	6	6	6	4	6	5	36	2nd
*Tamarindus indica*	4	3	5	5	3	2	4	26	4th
*Combretum molle*	5	4	5	5	5	4	6	34	3rd
*Acacia tortilis*	6	5	6	6	6	5	6	40	1st
*Acacia nilotica*	1	2	3	2	2	5	2	17	5th
*Gardenia ternifolia*	2	1	2	4	2	3	1	15	6th

**Fig. 13:**
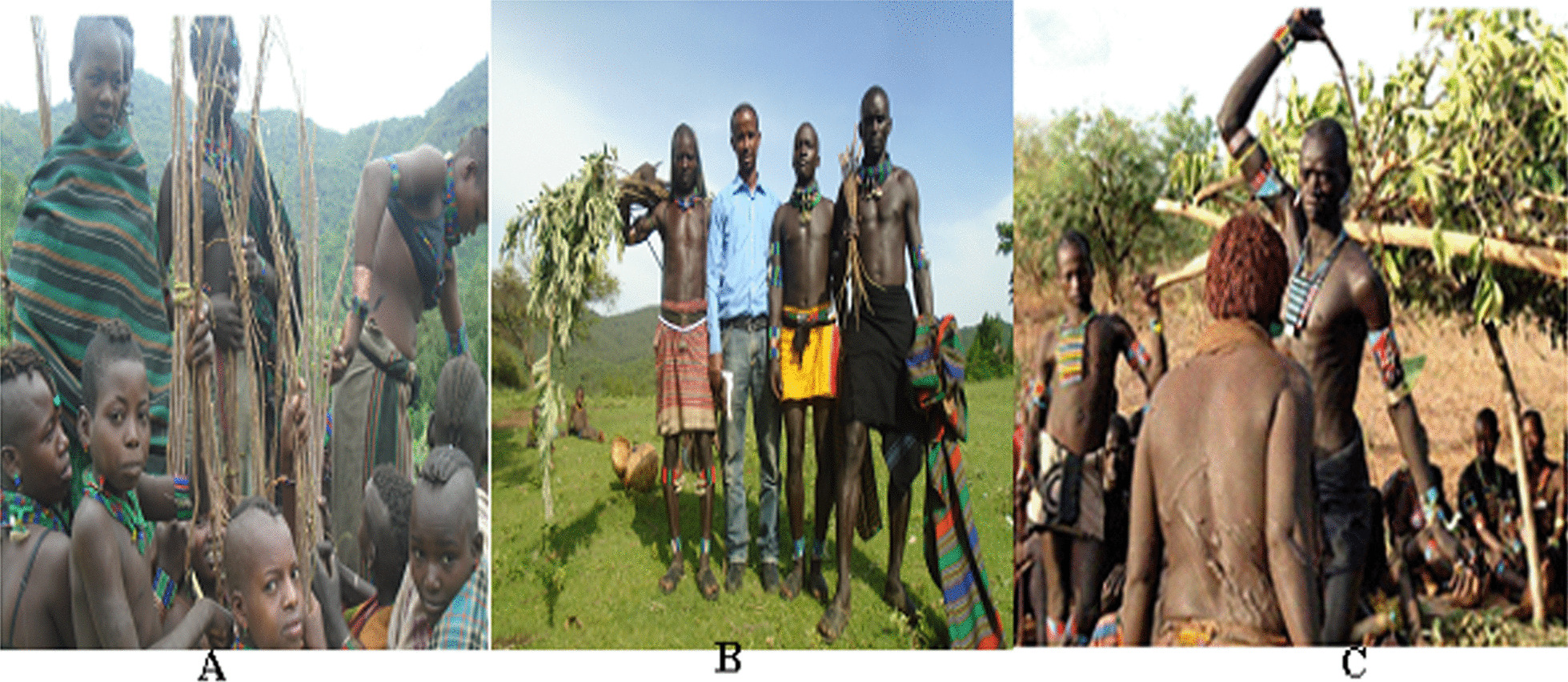
Youngsters collecting the stick/*’Michare’* (**A**), young males (Maaza) being ready for whipping the girl/*‘Anza’* (**B**) and the young male (Maaza) whipping the girl/*‘Anza’* (**C**) (photo by Melese Bekele 12, December 2020)

The findings of the study also indicated that the indigenous peoples in the study area have distinct perspectives on nature and natural resources in their surroundings. They have deeply ingrained cultural, religious, spiritual, and ceremonial norms regarding biodiversity in general, as well as specific plant species used for various purposes in the area. *Grewia ferruginea*, for instance, is one of the most culturally favored plant species for making the stick (locally known as ***Michare***) for whipping the young females (locally known as ***Anza***) by the young males (***Maaza***) who have recently jumped the bull-jump (***Equli-bulla***) during Hamar's most-known cultural ceremony known as ***Ivan-gadi*** (Fig. 13).

**Fig. 14:**
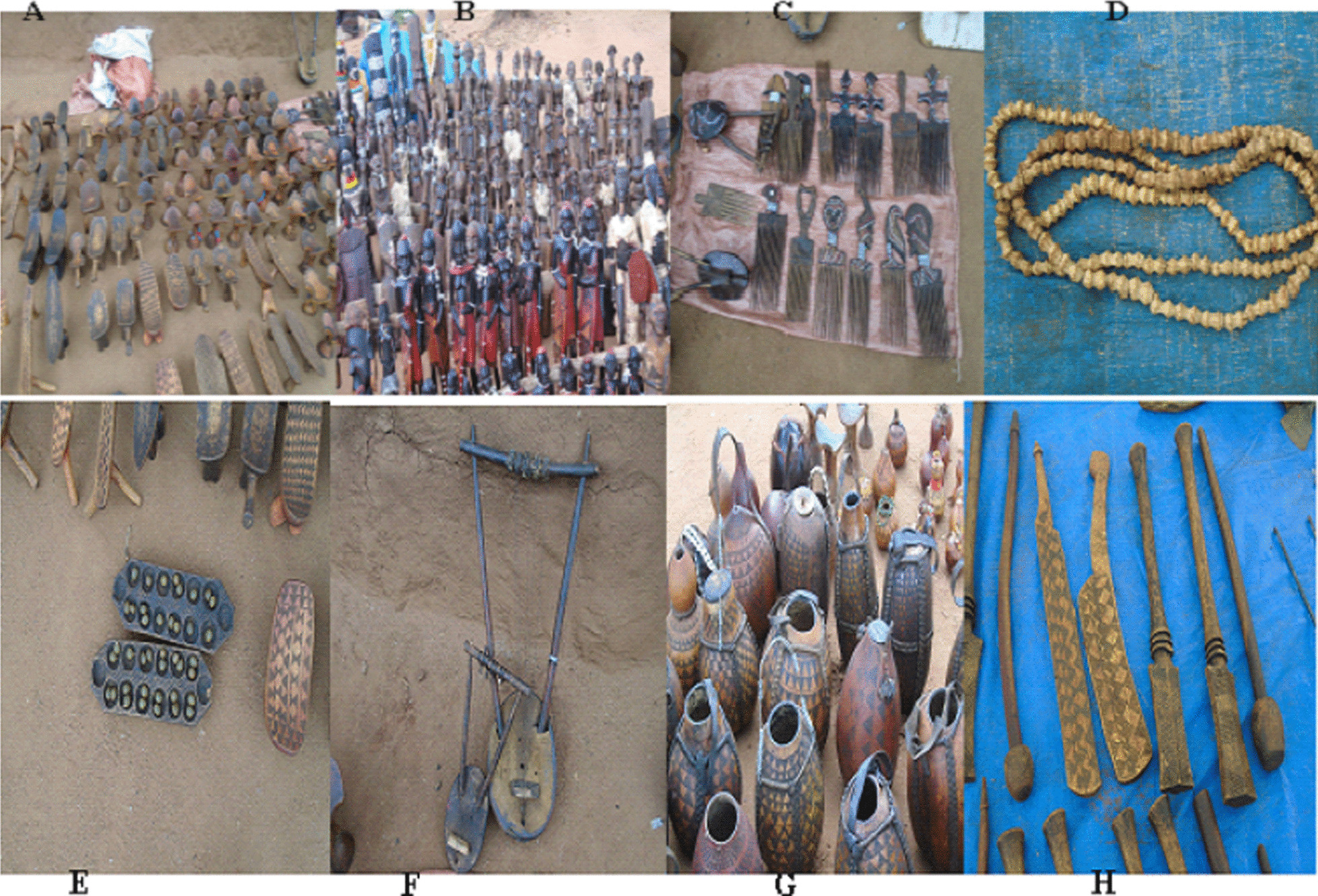
**A** *Borkota*, **B** *Hamarte*, **C** *Pasha*, **D** *Shikine*, **E** *Hamar gagie*, **F** *Ditie*, **G** *Gusi/Korsi* and **H** *Boko* (photo by Melese Bekele 20, December 2020)

About 86% of the plant species recorded from the study area were reported for their higher levels of use diversity depending on the indigenous culture and practice of the community. As a result, the study's findings revealed the presence of a hoarded indigenous practice of using plant products for household items, farming implements, arts, musical instruments, and beauties for the community and some of them were mentioned bellow from A–H with their local (Hamar) name (Fig. 14).

During the interview, informants mentioned that, various plant parts to be marketed in open markets to fulfill their economic and health needs. The market survey study also confirmed that selling and buying plant products is common in the study community.

**Fig. 15:**
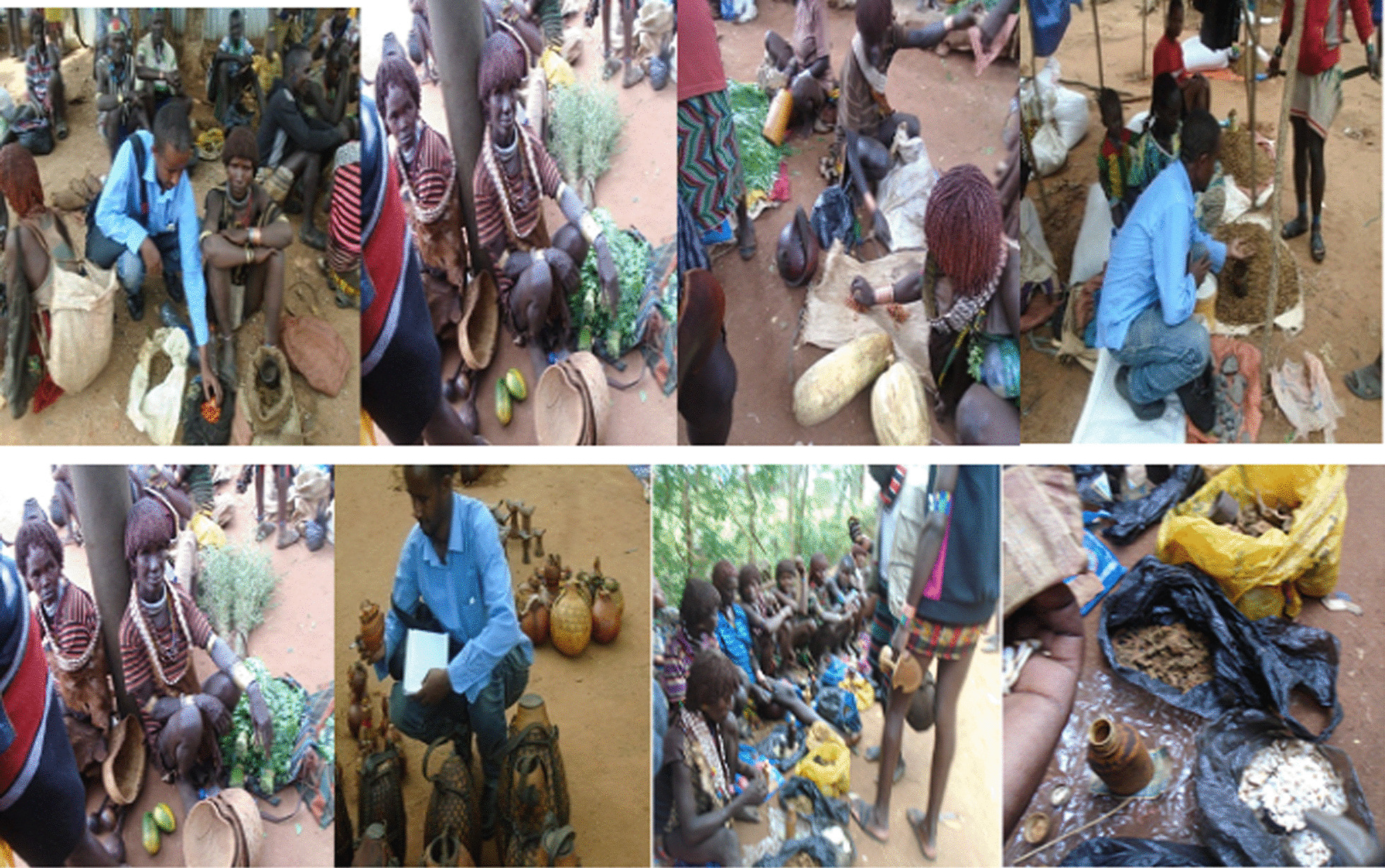
Venders selling various plant products in Dimeka Market (photo by Melese Bekele, 2019)

The informants claimed during the interview that various plant parts were sold in open markets to satisfy their needs in terms of both economics and health. The market survey study also indicates that buying and selling plants and plant parts is common in the study community. Plant products with multiple uses were also seen being sold in Dimeka open market. Herbal drugs, household utensils, and edible parts (fruits and leaves) were the most popular. About 55% of the plants sold in the market can be found at home gardens, fences, and semi-wild areas. On the other hand lower percentage of them (30%) specifically used for medicine and edible fruits, were collected from only the wild. The plant species and plant parts observed in the market for sale include: *Ximenia americana, Moringa stenopetala, Syzygium guineense, Gossypium arboretum, vigna unguiculata, Tamarindus indica, Dioscorea bulbifera, Lagenaria siceraria, Cucurbita pepo, Allium sativum, Amaranthus hybridus* and *Nicotiana tabacum* (Fig. 15).

### Cultural significance index (CSI)

The cultural significance index value considered for six most cited plant species revealed that *Grewia ferruginea* stands first having(CSI = 48) followed by *Olea europaea* subsp. cuspidata with a CSI value of 43.7(Table 14).

**Table 14:** The Cultural Significance Index (CSI) of some most cited plants: NIC = Number of informant citation, WV = Weighted Variables *i* = species management [managed (2) or non-managed (1)], *e* = Use preference [preferred (2) or not preferred (1)], *c* = Use frequency [frequently used (2) or rarely used (1)], CF = Correction factor [the number of informant citations for a given species divided by the number of informant citations for the most cited species]. (fod = fodder, foo = food, con = construction, fora = bee forage, furn = furniture, shad = shade and Cul/Rit = Cultural/Ritual use)

No	Plant species	WV	NIC	Specific uses							Sum	CF	CSI
				Fod	Foo	Con	Fora	Furn	Shad	Cul/Rit	(i * e * *c*)		
1	*Terminalia brownie*	*52*	(*i*)	1	1	1	1	1	1	1			
			(*e*)	2	1	2	2	2	2	2			
			(*c*)	2	1	2	2	2	2	2			
			(i * e * *c*)	4	1	4	4	4	4	4	25	0.76	19
2	*Piliostigma thonningii*	*47*	(*i*)	2	1	1	2	2	1	1			
			(*e*)	2	2	2	2	2	2	2			
			(*c*)	2	2	2	2	2	2	2			
			(i * e * *c*)	8	4	4	8	8	4	4	40	0.69	27.6
3	*Balanites aegyptiaca*	*54*	(*i*)	2	2	1	2	1	2	1			
			(*e*)	2	2	2	2	2	2	2			
			(*c*)	2	2	2	2	2	2	2			
			(i * e * *c*)	8	8	4	8	4	8	4	44	0.79	34.7
4	*Tamarindus indica*	*58*	(*i*)	1	2	1	2	1	2	1			
			(*e*)	2	2	2	2	2	2	2			
			(*c*)	2	2	2	2	2	2	2			
			(i * e * *c*)	4	8	4	8	4	8	4	40	0.85	34
5	*Grewia ferruginea*	*68*	(*i*)	2	1	2	2	1	2	2			
			(*e*)	2	2	2	2	2	2	2			
			(*c*)	2	2	2	2	2	2	2			
			(i * e * *c*)	8	4	8	8	4	8	8	48	1	48
6	*Olea europaea*	*62*	(*i*)	2	1	2	2	2	2	1			
			(*e*)	2	2	2	2	2	2	2			
			(*c*)	2	2	2	2	2	2	2			
			(i * e * *c*)	8	4	8	8	8	8	4	48	0.91	43.7

### Jaccard’s coefficient of similarity (JCS)

A thorough comparison of the current study's findings with those of other similar studies conducted in Ethiopia revealed a considerably greater record of medicinal plant species in the area. To realize the degree of species similarity, the result of Jaccard's coefficient of similarity was obtained. Thus, the current study and the study done in Blue Hora District had the highest degree of medicinal plant species resemblance (33%), followed by Amaro Woreda (26%), whereas the degree of similarity was lower with the study conducted in Northwestern Ethiopia and Konta Special Woreda (16%) (Table 15).

**Table 15:** Jaccard’s Coefficient of Similarity (JCS) b/n the current study and other similar studies conducted in Ethiopia

Study area/Author/s	*a*	*b*	*c*	JCS	%
Buska Mountain Range (present study)	145	–	–		
Afar Region, North Eastern Ethiopia [28]	103	28	42	0.24	24
East Gojjam Zone of Amhara region[29]	98	46	47	0.25	25
Meinit ethnic group of Ethiopia [30]	116	22	29	0.26	26
Debre Libanos Wereda, Central Ethiopia[31]	93	31	52	0.30	30
Blue Hora District/ [32]	81	45	64	0.33	33
Fiche District/Central Ethiopia [33]	79	89	66	0.28	28
Gera district, Ethiopia [34]	112	30	33	0.19	19
Konta Special Woreda, Southern [35]	106	84	39	0.16	16
South Omo/Southern Ethiopia [36]	104	53	41	0.20	20
Amaro Woreda [37]	101	15	44	0.26	26
Tigray Regional State/Northern Ethiopia [38]	95	35	50	0.27	27
Wayu Tuka District, West Ethiopia [39]	95	79	50	0.21	21
Wonago Woreda/South Ethiopia [40]	97	24	48	0.28	28
North Shewa Zone, Amhara Regional State[41]	75	85	70	0.30	30
Northwestern Ethiopia[42]	119	19	26	0.16	16
Western Ethiopia [43]	127	8	18	0.20	20
Ethiopian common medicinal plants [44]	97	32	48	0.27	27

Based on the analysis of variance (ANOVA), there was a positive correlation between informant’s age and ethnobotanical knowledge or a significant variation among the age categories at (*p* < 0.05) (Table 16).

**Table 16:** Correlation between age of informants and ethnobotanical knowledge

Multiple Comparisons						
Dependent Variable: Ethnobotanical knowledge						
Tukey HSD						
Age categories	Age categories	Mean difference	Std. error	Sig	95% confidence interval	
					Lower bound	Upper bound
20–35	36–65	− 3.333^*^	0.385	0.000	− 4.51	− 2.15
	66–88	− 7.333^*^	0.385	0.000	− 8.51	− 6.15
36–65	20–35	3.333^*^	0.385	0.000	2.15	4.51
	66–88	− 4.000^*^	0.385	0.000	− 5.18	− 2.82
66–88	20–35	7.333^*^	0.385	0.000	6.15	8.51
	36–65	4.000^*^	0.385	0.000	2.82	5.18

^*^ The mean difference is significant at the 0.05 level

### Traditional knowledge transfer system of the community

**Fig. 16:**
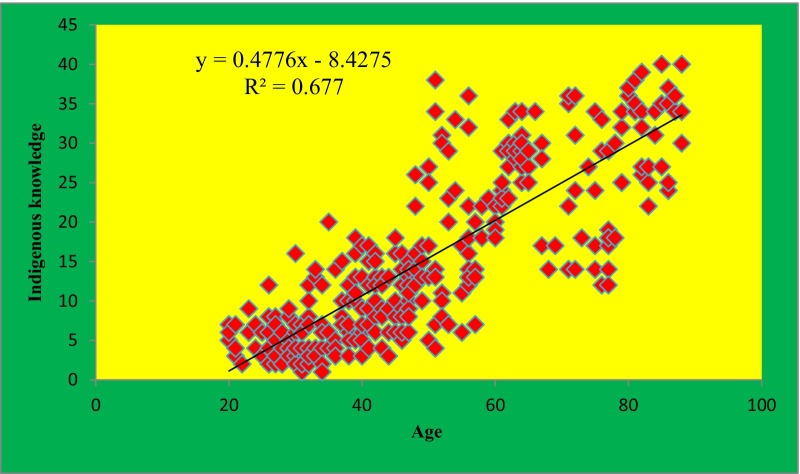
The correlation between age and indigenous knowledge in the community

In the Hamar community, the transfer of indigenous knowledge on the ethnobotanical plants use was most commonly by the words of mouth to their family members. The result of the study also indicated that the transfer system was the inheritance based by which most of the traditional healers transfer their knowledge being very selective based on the criteria they developed. Most usually, the closeness to the family, seniority in birth, ability to maintain secret, good conduct and gender (sometimes) were put as the main criteria for the selection. Accordingly, if the elder son/daughter become the candidate who can fulfill the criteria, he/she is given priority to have the knowledge. If he/she does not fulfill the requirement, the search for a hierarchal relationship to the family members is done to get the ideal nominee. Then during the transfer, the local community leaders become together, traditional ceremony (*Ivangadi* dance) is set, the transfer of traditional knowledge bounded by established rules and laws is done. The analysis of correlation between age and traditional knowledge also revealed that as age increases, the ethnobotanical knowledge of the community increases. i.e. there is a positive relationship between the age of informants and the indigenous knowledge they attain on use of plants (*r*^*2*^ = *0.6469* or r = 0.82) in the study area (Fig. 16).

### Major threats to the vegetation in the area

**Fig. 17:**
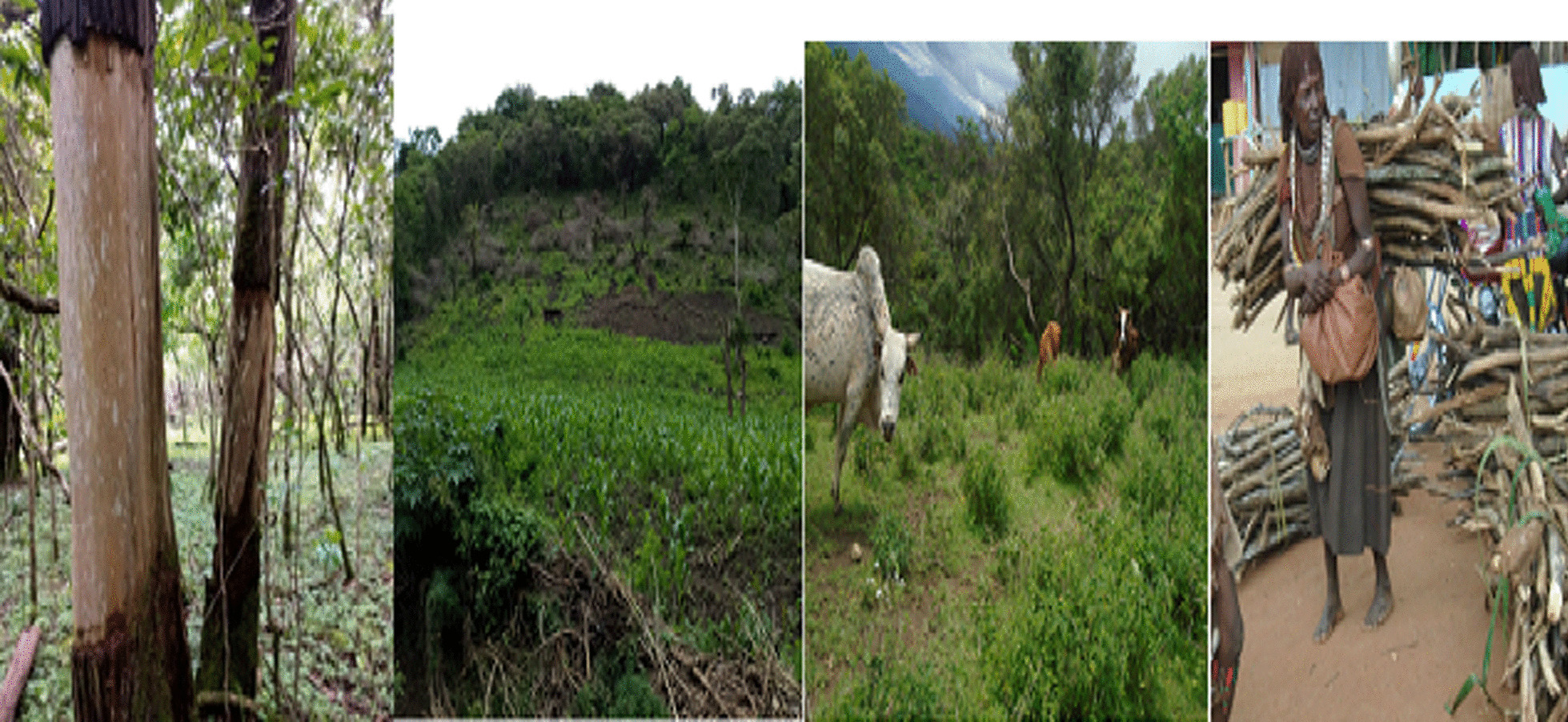
Major treats to plants in the study area (photo by Melese Bekele, 20 December 2020)

The informants reported the major threats to the vegetation of the study area, and their current status was also discovered during the field work. Debarking/ringing for beehive construction was identified as the most serious threat, followed by agricultural expansion and free grazing/overgrazing as the second and third most serious threats, respectively, in the area (Fig. 17).

A destructive effects ranking for major threats was conducted based on the informant's report as well as the field observation, taking into account their destructive status. As a result, ten key informants were chosen and asked to list and rank the factors on a scale of 1–7 (1-not/least destructive factor, 7-highly destructive factor). Accordingly, debarking (60 total score) came first, followed by agricultural expansion (54 total score) and free grazing (41 total score), with charcoal production being the least damaging threat factor in the area (Table 17).

**Table 17:** Major treating factors of the vegetation in the area

Major treats	K1	K2	K3	K4	K5	K6	K7	K8	K9	K10	Total	Rank
Abandoned fire	3	4	6	3	5	4	2	2	5	1	35	4th
Free grazing	6	5	4	1	6	3	4	3	6	3	41	3rd
Fuel wood extraction	2	3	2	5	7	5	1	6	1	2	34	5th
Debarking/ringing	7	6	5	7	2	7	6	7	6	7	60	1st
Agricultural expansion	5	7	7	6	1	6	7	4	7	4	54	2nd
Construction	4	1	3	2	4	1	3	5	3	5	31	6th
Charcoal production	1	2	1	4	3	2	5	1	2	6	27	7th

## Discussion

The indigenous people of the study area have a wealth of knowledge and understanding of the natural resources in their immediate surroundings. A total of 145 medicinal plant species used for human and livestock ailments were identified from the area. When compared to other studies conducted in Ethiopia, such as [35] (who recorded 120 medicinal plant species), [29] recorded 93 medicinal plant species, and [45] with 131 plant species, this study had a higher number of medicinal plant species. The greater record of medicinal plant species in the current study could be attributed to the local community's total reliance on plants for health problems, regardless of the type of ailment as well as the shortages of modern medical services in the study area. On the other hand, the number is lesser when compared to [33, 46] where respectively 155 and 173 medicinal plant species were recorded.

The popularity of shrubs may be due to their abundance and year-round availability, as opposed to trees that are selectively cut for various purposes, as well as the only seasonal appearance of herbaceous species after the rainy season in the area. The dominance of shrubs was also observed in other surveys conducted in various parts of the country [32, 36]. On the other hand, this finding disagrees with some other studies conducted elsewhere in Ethiopia where herbs were realized to be the dominant growth habits of the studied medicinal plant species [29, 30].

Among the plant families recorded for medicinal use in the area, Fabaceae was the most dominant family, followed by Asteraceae. Their dominance is most likely due to their ability to distribute a wider range, as well as the species richness and abundance in the study flora. This result is also consistent with the discoveries of a study conducted in other areas in Ethiopia where these families made the greatest contribution [39, 46–48]. In terms of plant parts used in the preparation of the remedies, leaves accounted for the greatest proportion when compared to other parts. This could be attributed to the higher perceived efficacy of fresh plant parts which could be lost upon drying, accessibility, ease of harvesting, simplicity of preparation, and chemical components it provides to treat the ailments. The finding is consistent with the results of other studies [49–52], where leaves were the dominant plant parts used for preparation of remedies.

According to an investigation of the conditions of the medicinal plants used to prepare remedies, about 78.8 percent of the medicinal plant parts were reported to be used freshly. This could be due to the close proximity of the source of the material (natural vegetation) to the majority of the healers in their environment, making it easier to prepare, as well as their belief that fresh plant parts are more effective than dry parts. Other studies found comparable results [32, 53]. The most commonly mentioned route of application was identified to be oral (57.7%) followed by dermal application (14.8%). This could be attributed to the rapid reaction of the medicine to treat the illness that the local healers realized through their longer experience. Similarly drinking was seen to be the most preferred method of administration for the remedies in the area. The current finding is in agreement with different other studies [13, 46]. Likewise, the most leading method of remedy preparation in the area was through crushing followed by squeezing, which is in coherent with the previous study [31, 54].

A considerable number of medicinal plant species (72) were used to treat only human ailments, followed by species useful to cure both human and animals diseases (45 species) and species applied to treat only livestock diseases (28). This confirms the local community's reliance on traditional medicine for health problems. The outcome is consistent with the reports of [36, 49]. Malaria was mentioned as the most common disease in the area, followed by wounds, snake bites, and stomach problems. The predominance of malaria could be attributed to the study district's hot climatic conditions. Generally, the dose of the remedy administered depends on the diseases treated, health status of the person/animal and the experience of the local healer. The highest ICF value (0.94) was recorded for reproductive problem category followed by the category of gastrointestinal disorder (ICF value of 0.92). According to [55], the high ICF values were important in identifying plants of an actual interest in the exploration for bioactive compounds.

The fidelity level values of medicinal plant species that are often utilized by the local community to treat one or a small number of illnesses are higher than those of less common species. Higher fidelity level may also be a sign of the plant's ability to effectively treat a certain ailment. Accordingly, the uppermost fidelity level (94.1%) was scored by *Phytolacca dodecandra* for treating rabies followed by *Albizia anthelmintica* with the fidelity level of (88.3%) for treating tapeworm and *Moringa stenopetala* with the fidelity level of 83.3% and used to treat cold/cough. Based on [24, 55], the fidelity level values could be used as a clue to identify medicinal plants of higher healing potential and those needed for further phytochemical and bioactive materials studies. In the preference ranking of different species used to cure malaria in the area, *Vepris dainellii* stood at the first position showing the most preferred species for treating malaria in the community followed by *Vernonia amygdalina* and *Gardenia ternifolia.* On the other hand, the preference ranking exercise on the eight plant species used against the most commonly reported cattle diarrhea in the area depicted that *Protea gaguedi* scored the uppermost rank followed by *Amaranthus hybridus*. Hence, the greater performance ranking value of the documented medicinal plant species could enable the forthcoming phytochemical and pharmaceutical studies and the conservation activities in the area.

### Non medicinal uses

Based on the results of ranking and scoring of plant species in specific use categories evaluated, the relative importance of each species in various use categories ensured major benefits. Accordingly, the highest preference ranking of *Acacia mellifera* for local house construction could be the strength of its wood as well as a better availability of the species in the area Similarly, the plant species used for timber production in the area were also seen to be very selective and few in number because of their practical preference for strength and quality.

The preference ranking of the seven plant species used for timber production revealed that *Cordia africana* scored the top rank followed by *Juniperus procera*. On the other hand, in the case of forage/fodder plant species, one plant species could be preferred over the other based on the seasonal availability and the product (amount and quality of milk, butter and others) of the livestock after feeding the forage plant. For instance due to a highly erratic nature of rainfall in the dry lands of the study region, there happens a nearby disappearance of nutritious grasses at the season for which trees and shrubs are an essential part of the pastoral to feed their livestock, which was not the case during the rainy season. A practical preference of various plant species for livestock shade, bee foraging/honey production, house hold utensils, beehive hanging and ceremonial and spiritual values was also seen in the area. The selection of the species could be based on their quality for the respective purposes used. For instance, canopy cover, quality of the honey produced, strength of the wood, height of the tree to be seen and cultural acceptance were respectively used as basic criteria for the preference. The commonly observed selling and buying practice of the plant parts and their products in an open market in the area, witnesses that the utmost dependence of the community on the plants in their environment to their day to day life as well as the cultural acceptance of using these resources in the area.

According to [20], many relative cultural significance indices group the distinct uses of plant species mentioned by informants into major use categories. As a result, the cultural significance index of the most frequently cited plant species in the area was assessed for various major use categories. For a variety of purposes, this could be used to prioritize and identify the most important and preferred species in the study area. Hence, in the study area, *Grewia ferruginea* (CSI = 48) was the most culturally significant species followed by *Olea europaea* subsp. cuspidata with a CSI value of 43.7. The highest degree of Jaccard’s coefficient of similarity (33%) was realized between the current study and the study done in Blue Hora District, Borana Zone [32] which could be attributed to the most related agro-ecological and climatic conditions in these study regions than the other areas compared.

### Major threats to the vegetation in the area

According to [56], the threats to biodiversity exceed the natural rate of regeneration and are primarily caused by habitat destruction, overharvesting, pollution, and the introduction of alien species. Based on the findings of this study, the major threats to medicinal plant species in particular, as well as to the vegetation of the Buska Mountain range in general, were debarking/ringing for beehive production, followed by agricultural expansion, free grazing/overgrazing, and abandoned fire, all of which were thought to be the most damaging in the area. The naturally erratic nature of the rainfall in the area, as well as the resulting frequent drought, combined with anthropogenic factors, endangers the biodiversity in the area along with the associated indigenous knowledge that requests for the community's conservation actions to be strengthened as soon as possible.

### Comparison with previous ethnobotanical studies in Ethiopia

Allium sativum L. was the most frequently used medicinal plant for Tuberculosis, internal parasites, and common cold treatment in the present study area. Likewise, Seyoum and Zerihun [31] reported use of this species for abdominal problem, common cold and snake bite in Debre Libanos Wereda, Central Ethiopia. On the other hand, Megersa et al. [39] reported it for malaria and headache treatment in east Welega zone of Oromia regional state, West Ethiopia. Wubetu et al. [42] reported the plants’ use in Dega Damot district, Northwestern Ethiopia for treatment of Evil eye.

Calpurnia aurea (Aiton) Benth.was the most frequently used plant and reported to be used against skin disease/scabies/and diarrhea in current study. Similarly, Lulekal et al. [55] reported the use of this species for tick infestation; helminthiasis and snake bite in Ankober District, North Shewa Zone, Amhara Region, Ethiopia; [31] from Central Ethiopia also reported the use of this plant for external parasites and abdominal pain. Tefera and Kim [57] mentioned the plant use for Gastritis, stomachache and toothache. Bekalo et al. [35], from the southern Ethiopia, reported the use of the species for treating snake bite. Its use also conveyed from West Ethiopia [39], for the treatment of Ascaris.

Stephania abyssinica (Dill. & A.Rich.) Walp. was the most commonly used medicinal plant in the present study area for coughing/pneumonia and amoeboid dysentery. Similar study from West Gojjam Zone, Amhara Region, Ethiopia [58] reported the use of this species for abdominal impelling, febrile illness and diarrhea. The species was also cited to treat wound [31], for common cold [39], to treat eye disease and amoeba [57].

Ocimum lamiifolium Hochst. ex Benth. was reported in the present study for the treatment of bloat/swelling and headache. Similarly, Seyoum and Zerihun from Debre Libanos Wereda [31] reported this plant for headache and cough. However, the species was reported for the fever and febrile illness in the study conducted in Tigray Regional State [38], for cough in Wonago Woreda [40] and Kassa et al.[59], from Sheka Zone reported it for the treatment of parasites.

The use of Phytolacca dodecandra L’Hér reported in the present study for the treatment of Ascaris, rabies and snake bite. In the study conducted in West Gojjam Zone [58], the plant was also mentioned to treat abdominal pain (swelling, vomiting), rabies and scabies. Megersa et al. [39], however reported this plant for Gonorrhea and liver disease, Lulekal et al.[55], reported the plant for treating ecto and endo-parasites.

In general, based on a comparison of our findings with earlier ethnobotanical studies in Ethiopia, a number of the plant species documented and reported for the treatment of various ailments in the current research community were reported in one way or another in various studies conducted in the country. The present study's findings did, however, also document some novel plant uses of medicinal plants that were only known to the current study community. Albizia anthelmintica (A.Rich.) Brongn., Protea gaguedi J.F.Gmel., Vangueria volkensii K.Schum., Solanum dasyphyllum, *Nicandra physalodes* (L.) Gaertn. and *Maerua angolensis* L. were utterly novel uses in our study area that had never been documented in other studies of a similar sort across the country. These plants' pharmacological properties are novel discoveries that have only been used in our study community. Thus, the species suggestion in this situation was used sparingly based on both actual field observations and contextualized source data from the community on the cultural worth and importance of plants. Comparing ethnobotanical indices across research and cultures can be difficult due to cultural differences, sample sizes, emic use categories, and a variety of other aspects; nonetheless, relevant constraints and considerations for the indices were taken into account during the evaluation.

The outcomes of the investigation also revealed that the research area was rich in ethnomedicinal plants and indigenous knowledge regarding their usage. Local travelers, for instance, demonstrated a strong understanding of these curative and lucrative medicinal plants in some of the study villages, as did local healers or traditional herbalists. Furthermore, the vegetation of the Buska Mountain range was known to be passed down from generation to generation by Hamar tribe leaders. As a result, the findings of this study will be critical in raising awareness and encouraging experience sharing both locally and among a broader audience, serving as a springboard for policies that prioritize public health and bioactivity. Similarly, it might be utilized as a foundation for future research on ethnomedicinal plants, stimulating phytochemical and pharmacological investigations as well as the sharing of practical experience of conservation efforts from the study community to the national level (Additional file 1).

## Conclusion

The people of the study area have accumulated indigenous knowledge of categorizing and using the vegetation in their surrounding area, as well as the rich plant resources, for various use categories. The findings of the study also publicized that the Buska Mountain range has relatively diverse plant species of medicinal use to treat human and livestock conditions.

More than 70 various human and livestock disorders have been identified as health issues in the area and are being treated by various plant species identified by the community. On the other hand, malaria was reported to be the most prevalent diseases in the area followed by wound, snake bite and tapeworm. Utmost reliance of the local community on the plant products of the vegetation in the area for other than medicinal values was also comprehended in the current study. However, currently, the biodiversity in the area in general, as well as the medicinal plant species in particular, are under a serious pressure and threats by most of anthropogenic factors combined with the current climatic conditions threatening the dry land areas in the country. As a result, the conservation activities of the local community should be reinvigorated and supported by scientific evidence based conservation actions as soon as possible.

## Supplementary Information

**Additional file 1.** Medicinal plants used for the treatment of human diseases. The file lists plant species used to treat human ailments, scientific and local name of plant species, plant part used, voucher number, methods of preparation and application.

## Data Availability

All the needed data collected for this study were analyzed and incorporated in this manuscript.
