# Supplementary material for: Ethnobotanical investigation of medicinal plants in Buska Mountain range, Hamar district, Southwestern Ethiopia
Source: J Ethnobiol Ethnomed. 2022 Sep 19;18:60. doi: 10.1186/s13002-022-00558-0 (PMC9484237; doi:10.1186/s13002-022-00558-0)
Supplement: Supplementary file 1 — Additional file 1. Medicinal plants used for the treatment of human diseases. The file lists plant species used to treat human ailments, scientific and local name of plant species, plant part used, voucher number, methods of preparation and application. [file 13002_2022_558_MOESM1_ESM.docx]

**Additional file 1:** Index 1: List of Medicinal plant species used in the study area (Habit: T-tree, S-shrub, H-herb, and C-climber)

| **Scientific name** | **Family** | **Local name** | **Habit** | **Voucher specimen no.** | **Parts**  **used** | **Preparation and application methods** | | | | | **Administration route** | | | **Disease treated** | |  |
| --- | --- | --- | --- | --- | --- | --- | --- | --- | --- | --- | --- | --- | --- | --- | --- | --- |
| *Hygrophila schulli*  *M.R.Almeida and S.M.Almeida* | Acanthaceae | Arbi | H | MB-058 | Root & stem | Dried stem and root used for home fumigating to avoid the harmful insects | | | | | Nasal | | | Insects biting and resulting diseases | |  |
| *Hypoestes aristata (Vahl) Sol. ex Roem. & Schult.* | Acanthaceae | Busente | H | MB-048 | Root | Crushed root for gastrointestinal disorder | | | | | Oral | | | Stomach problem | |  |
| *Barleriaeranthemoides R.Br. ex C.B.Clarke* | Acanthaceae | Chakidesha | H | MB-060 | Fresh leaf | Pounded, boiled with water, filtered and drunk orally | | | | | Oral | | | Evil eye | |  |
| *Justicia schimperiana T.Anderson* | Acanthaceae | Guset | S | MB-137 | Leaf | Leaf powder is used for asthma and the chewed root/ leaf juice used for tonsillitis | | | | | Nasal /oral | | | Asthma and tonsillitis | |  |
| *Acacia mellifera (Vahl) Bosc* | Fabaceae | Dhita | T | MB-033 | Root/bark | The crushed & powdered root/ bark is given orally for Tapeworm | | | | | Oral | | | Tapeworm | |  |
| *Allium sativum L.*. | Alliaceae | Tsemi  shinkurt | H | MB-039 | Bulb/rhizome | Grinded bulbs mixed with honey and taken for a week | | | | | Oral | | | Tuberculosis internal parasites, Common cold | |  |
| *Achyranthes aspera L.* | Amaranthaceae | Gotena | H | MB-025 | Root | Decocted fresh root mixed with *Aullium sativum* and a tea cup amount given /applied through nose | | | | | Oral | | | Nasal infection/ bleeding | |  |
| *Amaranthus hybridus L.* | Amaranthaceae | Zapina/ Hada | S | MB-050 | Leaf | Leaf of *Amaranthus hybridus* is pounded and boiled with water and given to cattle | | | | | Oral | | | Animal diarrhea | |  |
| *Ozoroa insignis Delile* | Anacardiaceae | Salbana | T | MB-068 | Bark | Chopping the inner part of the bark, mixed with milk and boiled then drink half a glass of it daily for 3-5 days for Cold disease | | | | | Oral | | | cold and cough | |  |
| *Lannea fruticosa (Hochst. ex A.Rich.) Engl.* | Anacardiaceae | Dafi | T | MB-087 | Root | The root is crushed and scrubbed on the affected body part | | | | | Dermal | | | Wound or swelling | |  |
| *Catha edulis (Vahl) Endl.* | Celasteraceae | Chata | S | MB-038 | Leaf | The leaf is Chewed /crushed and swallowed for hypertension , diabetes and headache | | | | | Oral | | | Shivering and breathing problem in animals | |  |
| *Foeniculum vulgare Mill.* | Apiaceae | Menzu | S | MB-055 | Root | A cup of Pounded root solution is given for stomach ache in humans and cattle | | | | | Oral | | | Diabetes, hypertension and headache | |  |
| *Heteromorpha arborescens (Spreng.) Cham. & Schltdl.* | Apiaceae | Tilo | S | MB-057 | Leaf | The leaves of *Heteromorpha arborescens* and *Croton macrostachyus* crushed with water a liter of it is given orally for shivering animal | | | | | Oral | | | Shivering | |  |
| *Coriandrum sativum L.* | Apiaceae | Dimbilali | H | MB-010 | Leaf | Leaf crush leaf for intestinal disorder | | | | | Oral | | | Stomach problem | |  |
| *Carissa Spinarum G.Lodd.* | Apocynaceae | Akamba | L | MB-047 | Fruit & leaf | Crushed fresh fruit is tied on swollen foot/leaf crushed and mixed with water a cup of it is given to drink | | | | | Oral/Dermal | | | Helminthiasis /Cowdriosis / Tick borne disease | |  |
| *Adenium obesum (Forssk.) Roem. & Schult.* | Apocynaceae | Gurdo | T | MB-075 | Root | Root powder applied for stomach problem in cattle | | | | | Oral | | | Stomach problem | |  |
| *Colocasia esculenta (L.) Schott* | Araceae | Boyno | H | MB-139 | Stem/ bark | Pounding and homogenized with water then given orally to drink | | | | | Oral | | | Leech | |  |
| *Phoenix reclinata Jacq.* | Arecaceae | Meta | T | MB-094 | Leaf | The squeezed drop of leaf is applied into eye | | | | | Dermal | | | Eye disease | |  |
| *Asparagus africanus Lam.* | Asparagaceae | Mulo | C | MB-148 | Root | Decocted root mixed with water and allowed to drink with 2 jug per day | | | | | Oral | | | For urine blocking in cattle | |  |
| *Asparagus officinalis L.* | Asparagaceae | Mulo | S | MB-149 | Whole part | Crushed fresh all parts of the plant mixed with water and given to drink | | | | | Oral | | | Coccidiosis/ bloody diarrhea in animals | |  |
| *Laggera pterodonta (DC.) Sch.Bip. ex Oliv.* | Asteraceae | Dampona | H | MB-144 | Leaf | Body brushed by leaf and also Crushed and mixed with water and external body wash | | | | | Dermal | | | Skin allergic reaction and evil spirit | |  |
| *Artemisia abyssinica (Schultz Bip.)* | Asteraceae | Nektri | H | MB-110 | Leaf | Fresh leaves grinded with *Allium sativum* is taken orally with food for gastrointestinal disorder | | | | | Oral | | | Stomach problems | |  |
| *Bidens pilosa L.* | Asteraceae | Achinti | S | MB-093 | Leaf | Squeezed fresh leaves juice is inhaled through nasal opening | | | | | Nasal | | | Nasal bleeding | |  |
| *Aspilia kotschyi (Sch.Bip. ex Hochst.) Oliv.* | Asteraceae | shukshimit | H | MB-072 | Leaf | Leaf of *Aspilia kotschyi* is smashed and 1-2 drops taken orally / rubbed externally | | | | | Oral/nasal/ dermal | | | Evil eye | |  |
| *Dioscorea alata L.* | Dioscoreaceae | Kotene | C | MB-080 | Tuber | Eaten for diabetes | | | | | oral | | | Diabetes | |  |
| *Spilanthes uliginosa Sw.* | Asteraceae | Wuswuso | H | MB-105 | Leaf, root and flower | Leaf and flower for tonsils and root for toothache | | | | | Oral | | | Tonsils and toothache | |  |
| *Vernonia amygdalina*  *Delile* | Asteraceae | Gera | H | MB-100 | Leaf | Leaf juice for wound and skin inflammation | | | | | Oral and dermal | | | Wound and Malaria, skin inflammation | |  |
| *Tagetes minuta L.* | Asteraceae | Ankadesh | S | MB-066 | Leaf | The chopped and grounded leaves filtrate and taken orally for diarrhea and vomiting | | | | | Oral | | | Diarrhea and vomiting | |  |
| *Ageratum conyzoides L.* | Asteraceae | Achinti | H | MB-027 | Leaf | Crushed leaf juice is applied through nose | | | | | Dermal | | | eye disease | |  |
| *Conyza sumatrensis (Retz.) E.Walker* | Asteraceae | wiso | H | MB-127 | Leaf | The leaf is crushed, decocted and taken a cup /glass of it given orally for diarrhea | | | | | Oral | | | Diarrhea | |  |
| *Balanites rotundifolia*  *(Tiegh.) Blatt.* | Balanitaceae | Kuz | T | MB-011 | Root | The fresh root chopping/ Crushed boiled with goat meat is taken for Gastro intestinal illness and intestinal parasites | | | | | Oral | | | Gastro intestinal illness and intestinal parasites | |  |
| *Balanites aegyptiaca (L.) Delile* | Balanitaceae | Dumuko | T | MB-092 | Bark | The chopped bark solution is given (a cup of for human) & a litter of it for livestock for about four to five days | | | | | Oral | | | Rabies | |  |
| *Stereospermum kunthianum Cham.* | Bignoniaceae | Zolpo | T | MB-097 | Leaf | The chopped root mixed with water and taken orally for abdominal pain | | | | | Oral | | | Abdominal problem | |  |
| Ehretia cymosa Willd. ex Roem. & Schult. | Boraginaceae | Darfi | S | MB-143 | Leaf | Smashed leaf sap of *Ehretia cymosa is taken* orally for overall body pain | | | | | Oral | | | Body pain | |  |
| Cordia sinensis Lam. | Boraginaceae | Malandongo | T | MB-012 | Root | Crushed fresh root mixed with water is taken for respiratory infection / tuberculosis | | | | | Oral/ nasal | | | Respiratory infection / tuberculosis | |  |
| Cynoglossum amplifolium Hochst. ex A.DC. | Boraginaceae | Yedena | S | MB-021 | Root/leaf | Crushed/ chopped leaf / root with water is taken in small amount for chronic fever in human | | | | | Oral | | | Fever | |  |
| Cordia africana Lam. | Boraginaceae | Asho | T | MB-152 | Root | The powder of the root is applied on the affected body part for itching | | | | | Dermal | | | Itching | |  |
| Lepidium sativum L. | Bracicaceaea | Fetso | H | MB-020 | Seed | Grinded seed powder is used to treat stomach disorder | | | | | Oral | | | Stomach disorder | |  |
| *Brassica carinata*  *A.Braun* | Brassicaceae | Gomen | HERB | MB-031 | Fruit | Grinded dry seed and/Crushed leaf mixed with boiled water and allowed to drink | | | | | Oral | | | Common cold and coughing | |  |
| *Boswellia neglecta*  *S.Moore* | Burseraceae | Ala | T | MB-098 | Bark exudates | The dried, burned and the smoke inhaled | | | | | Nasal | | | Evil eye | |  |
| Opuntia ficus-indica (L.) Mill. | Cactaceae | Dompo | S | MB-044 | Leaf | The leaf latex is used for drying the wound | | | | | Dermal | | | Wound | |  |
| Cadaba farinosa Forssk. | Capparidaceae | Lagee | S | MB-095 | Leaf | The fresh leaf is crushed and the juice is taken orally for malaria | | | | | Oral | | | Malaria | |  |
| Capparis tomentosa Lam.  Maerua angolensis L. | Capparidaceae  Capparidaceae | Mallo  Kadi | S  T | MB-014  MB-08 | Bark  Leaf | Crushed powder of the bark with hot water is taken orally for evil eye  Fresh leaf of it is cooked and eaten for dry stomach | | | | | Oral  Oral | | | Evil eye  Abdominal problem | |  |
| Carica papaya L. | Caricaceae | Papye | T | MB-0224 | Fruit | The ripe fruit eaten in the morning for abdominal dryness | | | | | oral | | | Abdominal problem | |  |
| Chenopodium procerum Hochst. ex Moq. | Chenopodiaceae | Ankidesha | H | MB-079 | Leaf/ stem | Leaf juice taken for stomach disorder, to pursuit ants , cleaning/ brushing animals body to avoid harmful insects biting the animal | | | | | Oral & Dermal | | | Helminthiasis /Cowdriosis / Tick borne disease and stomach disorder | |  |
| Combretum aculeatum Vent. | Combertaceae | Zurguma | T | MB-051 | Leaf | Fresh leaves chewed and the juice is swallowed | | | | | Oral | | | Abdominal problem | |  |
| Terminalia brownii Fresen. | Combertaceae | Ara | T | MB-042 | Bark | Inner part of the bark is chopped and the juice in taken orally | | | | | Oral | | | Jaundice/ intestinal problem | |  |
| Combretum collinum Fresen. | Combertaceae | Didiso | T | MB-082 | Leaf | The crushed leaf / roots mixed with water is given orally | | | | | Oral | | | Blackleg | |  |
| Combretum molle R.Br. ex G.Don | Combretaceae | Sebe | T | MB-141 | Root | steam fumigating the patient | | | | | Nasal | | | Evil eye | |  |
| Ipomoea kituiensis Vatke | Convolvulaceae | Gali | S | MB-129 | Leaf | Rubbing the body of restless anima/ human children with fresh leaf of *Ipomoea kituiensis* makes calm the animal / child | | | | | Dermal | | | Restlessness | |  |
| Kalanchoe lanceolata (Forssk.) Pers. | Crassulaceae | Gaya tampo | H | MB-140 | Leaf and Root | The leaf juice of *Kalancboe citrine* used for skin inflammation and allergic and the root juice is used against tapeworm | | | | | Oral/ dermal | | | Inflammation and allergic fever and tapeworm | |  |
| *Allophylus abyssinicus (Hochst.) Radlk.* | Sapindaceae | selal | S | MB-061 | Leaf/bark | A cup of crushed, decoction drunk for a week | | | | | Oral | | | Nephritis | |  |
| *Momordica foetida Schumach.* | Cucurbitaceae | Hasha | C | MB-125 | Root | Squeezed and homogenized with boiled water and given orally | | | | | Oral | | | Rabies | |  |
| *Lagenaria siceraria (Molina) Standl.* | Cucurbitaceae | Gusi | C | MB-037 | Root | Pounded dried root and mixed with cold water is given orally | | | | | Oral | | | Swelling / Cancer | |  |
| *Cucumis ficifolius A.Rich.* | Cucurbitaceae | Methi | C | MB-138 | Leaf | Pounded leaf of Cucumis *ficifolius* mixed with boiled water and a cup of it is taken orally for febrile illness | | | | | Oral | | | Febrile illness | |  |
| Cucurbita pepo L. | Cucurbitaceae | Bota | C | MB-147 | Leaf and seed | Seed for tapeworm and ascaris and the crushed leaf boiled with water and drunk a cup of it for a week used to treat coughing | | | | | oral | | | Tapeworm/ ascaris and coughing | |  |
| Juniperus procera Hochst. ex Endl. | Cupressaeae | Zizibe | T | MB-103 | Leaf | Crushed fresh leaf and mixed with water , filtered to drink and wash | | | | | Oral and Dermal | | | Ecto-parasites and endo-parasites | |  |
| Sansevieria ehrenbergii(Schweinf.) | Dracaenaceae | Alko | S | MB-088 | Leaf | Pounded fresh leaf juice is applied on the wound | | | | | Dermal | | | Wound | |  |
| Euclea racemosa L. | Ebenaceae | Kunsi | S | MB-049 | Bark | Bark powder is applied for Tina ( qakucha) | | | | | Tina | | | Dermal | |  |
| Euphorbia tirucalli L. | Euphorbiaceae | Tuzi | S | MB-085 | Shoot/ bark | Milk of the shoot/ bark applied on the woded part of the body | | | | | Dermal | | | Wound/ Swelling | |  |
| Tragia brevipes Pax | Euphorbiaceae |  | S | MB-123 | Root and leaf | Grinded root mixed with water and given orally | | | | | Oral | | | Parasitic leech and Dermatophilosis | |  |
| Croton macrostachyus Hochst. ex Delile | Euphorbiaceae | Bata | T | MB-017 | Leaf and root | Crushed root mixed with water given orally for stomach problem for animals, fire heated leaf steamed/ brushed the affected body for both animals and human , leaf juice for wound cure | | | | | Oral, nasal and dermal | | | Stomach disorder , muscle pain, fever, head ache | |  |
| Clutia lanceolata Forssk. | Euphorbiaceae | Wolsho | S | MB-151 | Leaf | The chopped leaf juice is given orally/ nasally to the cattle for bloody diarrhea | | | | | Oral /nasal | | | Cattle bloody diarrhea | |  |
| Euphorbia hirta L. | Euphorbiaceae | shudi | H | MB-122 | Leaf | The crushed leaf is rubbed on the affected body part | | | | | Dermal | | | Skin disease | |  |
| *Ricinus communis L.* | Euphorbiaceae | Ati | S | MB-026 | Seed/ leaf | Grinded seed / Crushed leaf mixed with water given orally | | | | | Oral | | | To remove a retained placenta | |  |
| Acacia tortilis (Forssk.) Hayne | Fabaceae | Sewute | T | MB-117 | Fresh leaf | Fresh leaf chopped and the juice isgive orally / fresh leaf is given to feed to goats intestinal parasites | | | | | Oral | | | To remove the goats intestinal parasites | |  |
| Millettia ferruginea (Hochst.) Hochst. ex Baker | Fabaceae | Zagi | T | MB-121 | Root | Crushed and decocted root dried mixed with boiled water , filtered and drunk | | | | | Oral | | | Trypanosomiasis | |  |
| Albizia anthelmintica (A.Rich.) Brongn. | Fabaceae | Rumbe | T | MB-013 | Leaf | Crushed fresh leaf/bark and mixed with water and given orally | | | | | Oral | | | Cough/ Tuberculosis & Tapeworm | |  |
| Calpurnia aurea (Aiton) Benth. | Fabaceae | Doferenda | S | MB-064 | Leaf | Leaf of *Calpurnia aurea* is pounded with *Croton macrostachyus* leaf and homogenized with water the skin disease /scabies / on the animals , seed and leaf powder taken for diarrhea | | | | | Dermal & oral | | | Skin disease /scabies / and diarrhea | |  |
| *Erythrina brucei Schweinf.* | Fabaceae | Beri | T | MB-107 | Bark and leaf | Bark of *Erythrina brucei* pounded with leaf of *Teclea nobilis* mixed with water and a glass of the solution is given orally and fresh leaves are pounded, squeezed and the juice is added to the eye. | | | | | Oral | | | Swelling and Eye disease | |  |
| *Crotalaria incana L.* | Fabaceae | Shashembe | S | MB-101 | Leaf | Crushed leaf of *Crotalaria incana* is applied on the swelled body part | | | | | Dermal | | | Swelling | |  |
| Indigofera hirsuta L. | Fabaceae | Erbo | S | MB-063 | Root | Chopped root mixed with water and given orally to drink | | | | | Oral | | | Milk problem in cows/ to improve milk production | |  |
| Ormocarpum trichocarpum (Taub.) Engl. | Fabaceae | Moshke | S | MB-111 | Leaf | Crushed/ chopped leaf with water is applied on swollen body part | | | | | body swelling / dermal | | | Swelling | |  |
| Senna occidentalis (L.) Link | Fabaceae | Tekera | S | MB-119 | Root | root powder for snake bite | | | | | Dermal | | | Snake bite | |  |
| Pterolobium stellatum (Forssk.) Brenan | Fabaceae | Gorda | S | MB-146 | Leaf and root | Root and leaf smock is applied for epilepsy | | | | | Nasal | | | Epilepsy | |  |
| Tamarindus indica L. | Fabaceae | Roke | T | MB-106 | Fruit | The fruit dissolved in the water and taken orally against askaris | | | | | Oral | | | Ascaris | |  |
| Caylusea abyssinica (Fresen.) Fisch. & C.A.Mey. | Resedaceae | Amoche | H | MB-145 | Leaf,/root | A glass of crushed decoction is drunk for epilepsy | | | | | Oral | | | Epilepsy | |  |
| *Acacia nilotica (L.) Willd. ex Delile* | Fabaceae | Golel | T | MB-133 | Bark | Chewed / crushed bark juice is used for stomach problem | | | | | Oral | | | Stomach problem | |  |
| *Indigofera arrecta Hochst. ex A.Rich.* | Fabaceae | Rigima | S | MB-134 | Root | The crushed root juice is swallowed for stomach problem | | | | | Oral | | | Stomach problem | |  |
| Geranium aculeolatum Oliv. | Geraniaceae | wuseno | H | MB-120 | Leaf | Chopped leaf is rubbed on the wound | | | | | Dermal | | | Anthrax | |  |
| Leucas martinicensis (Jacq.) R.Br. | Lamiaceae | Kochi | H | MB-091 | Leaf | Crushed leaf mixed with cold water and filtered and taken orally for haemorroids and tonslits | | | | | Oral | | | Hemorrhoids and tonsils | |  |
| Ocimum lamiifolium Hochst. ex Benth. | Lamiaceae | Busenti | S | MB-035 | Leaf | Crushed and rinsed with water given to drink/squeezed with in the nose | | | | | Oral | | | Bloat/ swelling and headache | |  |
| Ocimum americanum L. | Lamiaceae | basobili | S | MB-096 | Leaf | Grinded / Crushed fresh leaf mixed with Allium sativum , Capsicum frutescens and water is taken orally | | | | | Oral | | | Stomach disorder | |  |
| Ocimum basilicum L. | Lamiaceae | Bakira | S | MB-135 | Leaf | Grinded / Crushed fresh leaf mixed with Allium sativum , Capsicum frutescens and water is taken orally ,the shoot is also used to fumigate containers of milk | | | | | Oral and Dermal | | | Stomach disorder | |  |
| Leonotis ocymifolia (Burm.f.) Iwarsson | Lamiaceae | Deisha | H | MB-069 | Leaf/ stem | Squeezed fresh leaf/stem homogenized with water and given a tea cup amount to drink daily for 2-3 days | | | | | Oral | | | Blackleg/Anthrax | |  |
| Ocimum urticifolium Roth | Lamiaceae | kultub | S | MB-041 | Leaf | Leaf of *Ocimum urticifolium* and Croton macrostachyus are crushed together and taken in little amount orally or fumigated for fever/ Febrile illness | | | | | Oral / nasal | | | Fever/ Febrile illness | |  |
| *Ocimum gratissimum subsp.* | Lamiaceae | Busenti | S | MB-052 | Leaf | Crushed and rinsed with water and a cup of it taken orally/ Smashed leaf juice dropped with fingertip to the nose for headache | | | | | Oral/ nasal | | | Headache, malaria, sick eye , and fever | |  |
| Premna schimperi Engl. | Lamiaceae | Danguso | S | MB-130 | Root | Chopped root soaked in warm water and the filtrate is applied topically to treat tick on animals | | | | | Oral | | | ticks and skin infection on cattle | |  |
| Plectranthus longipes Baker | Lamiaceae | Pare | S | MB-045 | Leaf | Leaf soaked in hot water and drunken | | | | | Oral | | | Evil eye | |  |
| *Salvia nilotica Murray* | Lamiaceae | Bishdsha | H | MB-053 | Root | A chopped root is mixed with water and the sediment applied on the affected part | | | | | Dermal | | | Wound | |  |
| Nuxia congesta R.Br. ex Fresen. | Loganiaceae | Hali | S | MB-124 | Leaf | The fresh leaves of the plant are pounded with *Allium sativum* and boiled then a glass of it is drunk for cold disease | | | | | Oral | | | Cold disease | |  |
| Buddleja polystachya Fresen. | Loganiaceae | Meste | S | MB-022 | Leaf & bark | Hand smashed and the extracted juice is applied in nose for menstrual problem | | | | | Nasal | | | Menstrual problem | |  |
| Sida rhombifolia L. | Malvaceae | Goldoda | S | MB-102 | Root & bark | Crushed bark/ root mixed with boiled water and a cup of it given daily for 2- 3 days for intestinal parasites /Ascaries | | | | | oral | | | Ascaries | |  |
| Azadirachta indica A.Juss. | Meliaceae | Nimi | T | MB-028 | Leaf | Powdered leaf is mixed with water and given nasally to the animal affected by blackleg | | | | | Nasal | | | Blackleg / swelling | |  |
| Stephania abyssinica (Dill. & A .Rich.) Walp. | Menispermaceae | Bini | C | MB-065 | Leaf | Crushed leaf / root mixed with water and taken orally to cure amoeboid dysentery | | | | | Oral | | | Coughing / pneumonia and amoeboid dysentery | |  |
| Moringa stenopetala (Baker f.) Cufod.  Ziziphus mucronata Willd. | Moringaceae  Rhamnaceae | Kalanki  Anshal | T  T | MB-150  MB-05 | Leaf    Fruit |  | Leaf juice for cold and cough and for food value  Fruit is eaten for dry stomach and food value | | Oral  Oral | | | | | | cold and cough  Stomach disorder | |
| Ensete ventricosum (Welw.) Cheesman | Musaceae | Hakame(Arigna) | H | MB-083 | Rhizome and stem | Smashed and given to the animal | | | | | | Oral | | Retained placenta is removed | |  |
| Maesa lanceolata Forssk. | Myrsinaceae | Teri | S | MB-029 | Fruit & leaf | Squeezed fruit and juiced with lemon and applied by a fingertip on infected area / skin , crush leaf rubbed for Hemorrhoids & fruit powder for tapeworm | | | | | | Dermal and oral | | Mange, Tick infestation/Parasitic leech/Helminthiasis, Hemorrhoids & tapeworm | |  |
| Syzygium guineense (Willd.) DC. | Myrtaceae | Ochote | T | MB-030 | Bark | Bark powder mixed with water is used for intestinal disorder | | | | | | Intestinal disorder | | Oral | |  |
| Eucalyptus globulus Labill. | Myrtaceae | barzafa | T | MB-032 | Leaf | Inhalation of leaf steam is used as remedy for cough / cold and head ache | | | | | | Nasal and dermal | | Cough / cold & head ache | |  |
| Plantago lanceolata L. | Plantaginaceae | Herbi | H | MB-104 | Leaf | Chopping the leaf and a glass of the solution is given orally | | | | | | Oral | | Leech infection | |  |
| *Olea europaea L. subsp. cuspidata* | Oleaceae | Rimit | T | MB-109 | Shoot | Leaf extracted juice is poured on infected part or Pounded leaf homogenized with cold water and allowed the animal to drink it , back powder for tapeworm & also remedy for malaria | | | | | | Oral | | Mange/skin disease, tapeworm | |  |
| Jasminum abyssinicum Hochst. ex DC. | Oleaceae | Shakanti | C | MB-054 | Root | Crushed root mixed with water is used against tapeworm & wound dressing | | | | | | Tapeworm & wound | | Oral and dermal | |  |
| Phytolacca dodecandra L'Hér. | Phytolaccaceae | Wusakalpi | L | MB-099 | Root & leaf | The root powder is taken for stomach disorder/ ascaris/ crushed leaf mixed with water is taken for rabies and snake bite | | | | | | oral and dermal | | Ascaris , rabies and snake bite | |  |
| Pennisetum setaceum (Forssk.) Chiov. | Poaceae | Tuti | H | MB-089 | Leaf | The crushed leaf is mixed with water and given orally for snake bite | | | | | | Oral | | Snake bite | |  |
| Cymbopogon citratus (DC.) Stapf | Poaceae | woshmi | H | MB-112 | Leaf | crushed leaf mixed with boiled water taken for Common cold, Odor /spices and stomach complaints | | | | | | Oral | | Common cold and stomach complaints | |  |
| Podocarpus falcatus (Thunb.) Endl. | Podocarpaceae | Gurikunta | T | MB-086 | Fruit & root | Decocted dried root / fruit and tied or snorted the drops in to the nose or smoked/mixed with hot water the infected area for one week | | | | | | Nasal and Dermal | | Evil spirit | |  |
| *Securidaca longepedunculata Fresen.* | Polygalaceae | sengano | S | MB-078 | Root & bark |  | Root juice for intestinal problem, root steam for evil eye, and snake bite | | | Oral , nasal and dermal | | | | | Intestinal problem, evil eye and snake bite | |
| Rumex nepalensis Spreng. | Polygonaceae | Kachanti | H | MB-019 | Root | Root of *Rumex nepalensis* is pounded and taken with a cup of tea for stomach ache & teeth ache | | | | | | | Oral | Stomach problem & teeth ache | |  |
| Protea gaguedi J.F.Gmel. | Proteaceae | Kewush | T | MB-059 | Leaf | A liter of chopped leaf solution is given orally for diarrhea in cattle | | | | | | | Oral | Diarrhea | |  |
| Clematis simensis Fresen. | Ranunculaceae | Gurti | C | MB-062 | Flower | fresh crushed flower applied to nose for head ache | | | | | | | nasal | Head ache | |  |
| Rhamnus prinoides L'Hér. | Rhamnaceae | Gurdanti | L | MB-126 | Leaf | Leaf and fruit juice for cattle stomach disorder and for tonsil in human | | | | | | | Oral | Stomach disorder, tonsil | |  |
| Prunus africana (Hook.f.) Kalkman | Rosaceae | Gorfok | T | MB-118 | Leaf | A chopped leaf is applied on wound | | | | | | | Dermal | Wound | |  |
| Canthium pseudosetiflorum Bridson | Rubiaceae | Medhel | S | MB-136 | Leaf/bark | Leaf/ bark chopped with in water and juice is drunk orally | | | | | | | Oral | Malaria | |  |
| Gardenia ternifolia Schumach. & Thonn. | Rubiaceae | Gembela | T | MB-076 | Leaf | Crushed leaf mixed with water and taken a cup of it for Malaria | | | | | | | Oral | Malaria | |  |
| *Galiniera saxifraga (Hochst.) Bridson* | Rubiaceae | Sele | S | MB-113 | Bark | A chopped bark is mixed with water and given | | | | | | | Oral/ nasal | Cattle diarrhea | |  |
| Coffea arabica L. | Rubiaceae | Buno | S | MB-081 | Fruit | Chopped *Coffea arabica* seed is boiled with water and a cup of it is drunk for head ache and discomfort feeling | | | | | | | oral | Headache & discomfort | |  |
| Pavetta gardeniifolia Hochst. ex A.Rich. | Rubiaceae | shmbulo | S | MB-016 | Root | The fresh root is chewed for tooth ache | | | | | | | oral | Tooth ache | |  |
| Vangueria volkensii K.Schum. | Rubiaceae | Gara | S | MB-128 | Leaf/ root | The crushed leaf/ root juice mixed with cow milk is given orally | | | | | | | Oral | Epilepsy | |  |
| Citrus limon (L.) Osbeck | Rutaceae | Lomi | S | MB-074 | Fruit | Half a cup of squeezed juice take orally for stomach problems and tonsil | | | | | | | Oral | Stomach problems and tonsil | |  |
| Clausena anisata (Willd.) Hook.f. ex Benth. | Rutaceae | Teri | S | MB-034 | Bark & root | Crushed bark remedy for Malaria and root is chewed for tooth ache | | | | | | | Oral and dermal | Malaria and tooth ache | |  |
| Ruta chalepensis L. | Rutaceae | Tselti | S | MB-142 | Leaf | A cup of Smashed leaf of *Ruta chalepensis* mixed with water is taken orally for stomach ache and evil eye | | | | | | | Oral | Gastrointestinal disorder, evil eye | |  |
| Vepris dainellii (Pic.Serm.) Kokwaro | Rutaceae | Kena | S | MB-084 | Leaf/ bark | Chopped with water and the juice is drunk | | | | | | | Oral | Malaria and abdominal colic | |  |
| *Salvadora persica L.* | Salvadoraceae | Kerja | S | MB-108 | Root/stem/ bark | Root ,stem/ bark chewed, and juice kept in the mouth | | | | | | | Oral | Tooth problem / bleeding gum | |  |
| Osyris quadripartita Salzm. ex Decne. | Santalaceae | Kundlish | S | MB-131 | Leaf | The crushed leaf juice is applied topically on the affected part | | | | | | | Dermal | Body / skin ulcer | |  |
| Dodonaea angustifolia L.f. | Sapindaceae | Serko | S | MB-023 | Leaf & root | Dried leaves powder of *Dodonaea angustifolia* is used to drying wound and also applied for tooth ache | | | | | | | Oral | Wound and tooth ache | |  |
| Brucea antidysenterica J.F.Mill. | Simaroubaceae | Abala | S | MB-132 | Leaf | Crushed leaf mixed with water and squeezed in a cup and used for Amebiasis/ amebic dysentery/ | | | | | | | Oral | Amebiasis/ amebic dysentery | |  |
| Solanum incanum L. | Solanaceae | Geranti | S | MB-015 | Bark | A chopped bark is mixed with water and given | | | | | | | Oral | Snake bite | |  |
| Datura stramonium L. | Solanaceae | Atimdisha | H | MB-115 | Leaf | Rinsed by hand and applied on skin/Crushed and mixed with water and the filtrate given | | | | | | | Dermal | Ringworm /Cowdriosis | |  |
| Solanum dasyphyllum Schumach. & Thonn. | Solanaceae | Garanti | S | MB-070 | Leaf | The sup of the crushed leaf drunk for common cold and also used as food | | | | | | | Oral | Common cold and cough | |  |
| Nicandra physalodes (L.) Gaertn. | Solanaceae | Etindesh | H | MB-040 | Leaf | Applied for wound dressing/drying | | | | | | | dermal | Wound | |  |
| Nicotiana tabacum L. | Solanaceae | Tambo | S | MB-073 | Leaf | dried ,Crushed & powdered leaf of *Nicotiana tabacum* is mixed with water is given to cattle to expel leeches , | | | | | | | Oral and nasal | Leeches, head ache | |  |
| Withania somnifera (L.) Dunal | Solanaceae | Butambero | S | MB-116 | Leaf & bark | The crushed leaf mixed with water is taken nasally/ orally for head ache & Sudden sickness | | | | | | | Oral / nasal | Head ache & Sudden sickness | |  |
| Capsicum frutescens L. | Solanaceae | Barbar | H | MB-114 | Fruit | Grinded/Pulverized dried fruit and mixed with *Zinjiber officinale* and *Allium sativum* rinsed in distillated butter and a tea cup amount let to feed daily morning | | | | | | | Oral | Pneumonia / cough/ stomach disorder | |  |
| Dombeya torrida (J.F.Gmel.) Bamps | Stericuliaceae | Kami | T | MB-090 | Root | The root is grinded and pasted on affected part / the grinded/chewed root juice is swallowed | | | | | | | Oral | Snake bite | |  |
| Sterculia africana (Lour.) Fiori |  | Kaiyito | T | MB-036 | Bark & root | Root and bark smocked for evil eye &chewed for snake bite | | | | | | |  | Snake bite & evil eye | |  |
| Grewia villosa Willd. | Tialiaceae | Gergesho | S | MB-046 | Fruit | The fresh fruit is chewed and for intestinal parasites | | Oral | | | | |  | | |  |
| Grewia bicolor Juss. | Tiliaceae | Baraza | S | MB-077 | Leaf & root | Crushed and mixed with *Croton macrostachyus* cold water and filtered to drink or/and wash daily | | | | | | | Oral and Dermal | | Ecto-parasites and dried stomach in animals |  |
| Grewia ferruginea Hochst. ex A.Rich. | Tiliaceae | Baraza | S | MB-018 | Leaf & bark | Pounded and grinded leaf and bark of *Grewia ferruginea* is mixed with water and a glass of it is given to cattle to expel the retained placenta | | | | | | | Oral | | Retained placenta |  |
| *Lantana camara L.* | Verbenaceae | Metsi | S | MB-067 | Leaf | Fresh leaf is steam heated body brushed /rubbed and also leaf juice applied nasally for headache | | | | | | | Dermal and nasal | | Allergic reaction and head ache |  |
| *Verbena officinalis L.* | Verbenaceae | Gunidesh | H | MB-043 | Leaf | Leaf squeezed by hand and mixed with water and given orally | | | | | | | Oral | | Snake bite |  |
| *Cissus quadrangularis L.* | Vitaceae | Choko | C | MB-056 | Root/stem/ bark | Any part of it is crushed/chopped and tied on the neck of the cattle for evil eye | | | | | | | Dermal | | Evil eye |  |
| *Zingiber officinale Roscoe* | Zingiberaceae | jenjele | H | MB-071 | Rhizome | Decocted, homogenized in boiled water, mixed with *Allium sativum* and taken orally | | | | | | | Oral | | Abdominal problem |  |
